# Preliminary Evidence Suggests the Transcriptomic Response of Tricolored Bats to White‐Nose Syndrome Is Similar but Not Identical to That of Little Brown Bats

**DOI:** 10.1002/ece3.74588

**Published:** 2026-10-28

**Authors:** Robert E. Kwait, Evan A. Eskew, Maanasvi Balasubramanian, Sarah A. Gignoux‐Wolfsohn, Malin L. Pinsky, Brooke Maslo

**Affiliations:** ^1^ Department of Ecology, Evolution, and Natural Resources, Rutgers The State University of New Jersey New Brunswick New Jersey USA; ^2^ Institute for Interdisciplinary Data Sciences University of Idaho Moscow Idaho USA; ^3^ Department of Biological Sciences University of Massachusetts Lowell Massachusetts USA; ^4^ Department of Ecology & Evolutionary Biology University of California Santa Cruz Santa Cruz California USA

**Keywords:** evolution, gene expression, immunology, pathology, physiology, wildlife disease

## Abstract

Understanding the pathophysiology of emerging wildlife diseases can inform treatment, improve the projections of long‐term population trajectories, and reveal variation in population‐level impacts among species. Research on white‐nose syndrome (WNS) in hibernating North American bats has largely focused on a single highly susceptible species, the little brown bat ( *Myotis lucifugus* ). Another susceptible species, the tricolored bat ( *Perimyotis subflavus* ), has experienced population declines comparable to or greater than those of little brown bats but remains understudied. In particular, the immune response of tricolored bats to WNS has not previously been evaluated. Here, we conduct a preliminary analysis comparing part of the transcriptomic response of tricolored bats to WNS with the little brown bat response. We analyzed 14 tricolored and 13 little brown bat samples collected from Colossal Cave, Kentucky, USA in 2017, where WNS has been present since 2013. We also included 26 little brown bat samples collected from a nearby hibernation site in 1999, prior to WNS emergence. Lacking a proper pre‐WNS control for tricolored bats, we conducted pairwise differential expression analyses between the three sample groups and evaluated the overlap between results to infer part of the tricolored bat response to WNS. These initial results suggested that tricolored bats exhibited differential expression patterns characteristic of susceptible species, including large changes in gene expression still apparent at the end of the hibernation season. Differentially expressed genes were associated with immune, metabolic, and circadian functions, broadly resembling the response observed in little brown bats. However, there was variation in the specific genes involved between the two species. Overall, tricolored bats appear to mount a pronounced immune response to WNS that is similar, but not identical, to that of little brown bats.

## Graphical abstract

In this study, we evaluate gene expression of tricolored bats in response to white‐nose syndrome and compare it to that of little brown bats. We found that tricolored bats show evidence of a similar transcriptomic immune response to little brown bats, sharing pathways and some genes. However, there were also a lot of differences in the genes that were differentially expressed between the two species.

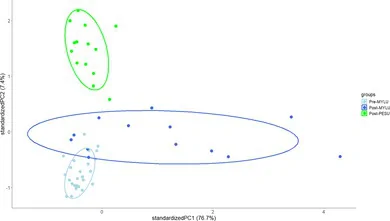

## 1 Introduction

The emergence of wildlife diseases has increased in recent decades and poses a major threat to biodiversity (Aguirre 2017; Marano et al. 2007; Wu et al. 2017). Several wildlife diseases have caused mass mortality events, drastic population declines, and even extinctions, including chytridiomycosis in amphibians (Kilpatrick et al. 2010; Scheele et al. 2019) and contagious facial tumor disease in Tasmanian devils ( *Sarcophilus harrisii* ) (McCallum et al. 2007; Pye et al. 2016). White‐nose syndrome (WNS) in hibernating bats is one of the most severe wildlife diseases in North America. It is caused by the invasive fungal pathogen, *Pseudogymnoascus destructans* (Lorch et al. 2011), which was introduced from Europe around 2006 (Blehert et al. 2009; Fischer et al. 2025; Hoyt et al. 2021; Leopardi et al. 2015; Zukal et al. 2016). During hibernation, *P. destructans* colonizes bat epidermal tissue, and host responses to infection trigger frequent torpor arousals and depletion of fat and water reserves (Cryan et al. 2010; Reeder et al. 2012; Verant et al. 2014; Warnecke et al. 2013). Population declines exceeding 90% have been documented in some bat species and continue to threaten long‐term population persistence (Cheng et al. 2021; Hoyt et al. 2021).

Despite the conservation importance of WNS, the pathophysiology of the disease has been detailed in relatively few species. Understanding host responses at the molecular level can inform conservation by helping to identify mechanisms of infection resistance or tolerance, improve projections of population trajectories, and develop effective treatment strategies. Wildlife hosts that are susceptible to disease often exhibit heightened and prolonged changes in gene expression activity relative to more resistant species: in a meta‐analysis of multiple wildlife disease systems, 73% of comparisons between susceptible and non‐susceptible host species revealed larger transcriptomic responses in the susceptible taxa, potentially reflective of ineffective pathogen control or immune system dysregulation (Eskew et al. 2021). However, patterns vary among disease systems, and the mechanisms driving these responses are not fully understood (Eskew et al. 2021). Within WNS research, transcriptomic responses have been extensively examined in only one susceptible host, the little brown bat ( *Myotis lucifugus* ) (Davy et al. 2020; Field et al. 2018; Lilley et al. 2019), which experienced declines exceeding 90% across its infected range (Cheng et al. 2021; Hoyt et al. 2021). Outside of little brown bats, big brown bats ( *Eptesicus fuscus* ) are the only North American species whose transcriptomic profile has been evaluated, and they are considered more resistant to WNS (Davy et al. 2020; Frank et al. 2014).

Little brown bats exhibit a transcriptional response to pathogen exposure consistent with patterns observed in other disease‐susceptible wildlife taxa (Eskew et al. 2021), characterized by large changes in gene expression that persist throughout most of the hibernation period (Davy et al. 2020; Kwait, Eskew, Pinsky, et al. 2025; Lilley et al. 2019). This response resembles typical mammalian defenses against fungal infection (Field et al. 2015; Moore et al. 2011; Whiting‐Fawcett et al. 2021), except that adaptive immunity appears constrained, likely due to the suppressed state of torpor (Lilley et al. 2017). Much of the immune response occurs during periodic arousals from torpor, while components of innate immunity remain active even while torpid (Field et al. 2018; Hoyt et al. 2021; Verant et al. 2014). While such immune activity would seem to be a beneficial response to infection, maintenance of elevated pathogen loads and persistence of immune activity throughout hibernation have the potential to result in immunopathology (Kwait, Eskew, Pinsky, et al. 2025; Lilley et al. 2019), as evidenced by systemic inflammation upon emergence from hibernation and immune activity outside areas of local infection (Davy et al. 2020; Meteyer et al. 2012). Little brown bat mortality is therefore thought to result primarily from physiological disruptions caused by fungal growth and increased torpor arousal frequency (Cryan et al. 2010; Verant et al. 2014; Warnecke et al. 2013).

The tricolored bat ( *Perimyotis subflavus* ) is another species severely affected by WNS. Population declines exceeding 90% have been documented, with disease impacts reported across 97% of surveyed hibernacula, on par with impacts on little brown bats (Cheng et al. 2021). Historically common throughout eastern North America, from eastern Canada to Colorado and south to Nicaragua (McCoshum et al. 2023; Medina‐Fitoria et al. 2015), tricolored bats are increasingly rare and have been proposed for listing as federally endangered in the United States (Ford et al. 2011; Huebschman 2019; McCoshum et al. 2023; Pettit and O'Keefe 2017). Despite the impacts of disease‐driven declines, the molecular and physiological responses of this species to WNS remain largely uncharacterized: tricolored bats produce antibodies against *P. destructans* and evidence from cell cultures suggests immune activity in response to *P. destructans* infection (Johnson et al. 2015; Lupfer et al. 2020), but other relevant details are unknown. Importantly, as one of the few susceptible species outside of the genus *Myotis*, tricolored bats may exhibit distinct responses to *P. destructans* infection.

Here we conduct a preliminary analysis to characterize the transcriptomic response of tricolored bats to *P. destructans* infection and compare it with that of little brown bats. We analyzed gene expression patterns in wing biopsy samples collected from wild bats at the end of hibernation, comparing little brown bats sampled before the emergence of WNS with little brown and tricolored bats sampled several years after disease establishment. To our knowledge, this study provides the first description of the transcriptional response to WNS in tricolored bats and represents one of the first comparative analyses of highly susceptible bat species.

## 2 Methods

Our dataset consisted of samples from both little brown and tricolored bats exposed to WNS, as well as naïve little brown bats. We analyzed 28 little brown bat samples collected from Cave Hollow Cave, Kentucky in March 1999 (Figure 1, Table A1). The pre‐WNS samples were 2 mm wing punches collected from torpid bats that were manually removed from the hibernaculum substrate. Samples were placed in 1.5‐mL tubes filled with DMSO and stored at −80°C. Because these samples were collected prior to the emergence of WNS (hereafter, pre‐WNS samples), we treated them as baseline, naïve gene expression patterns in unexposed little brown bats.

**FIGURE 1:**
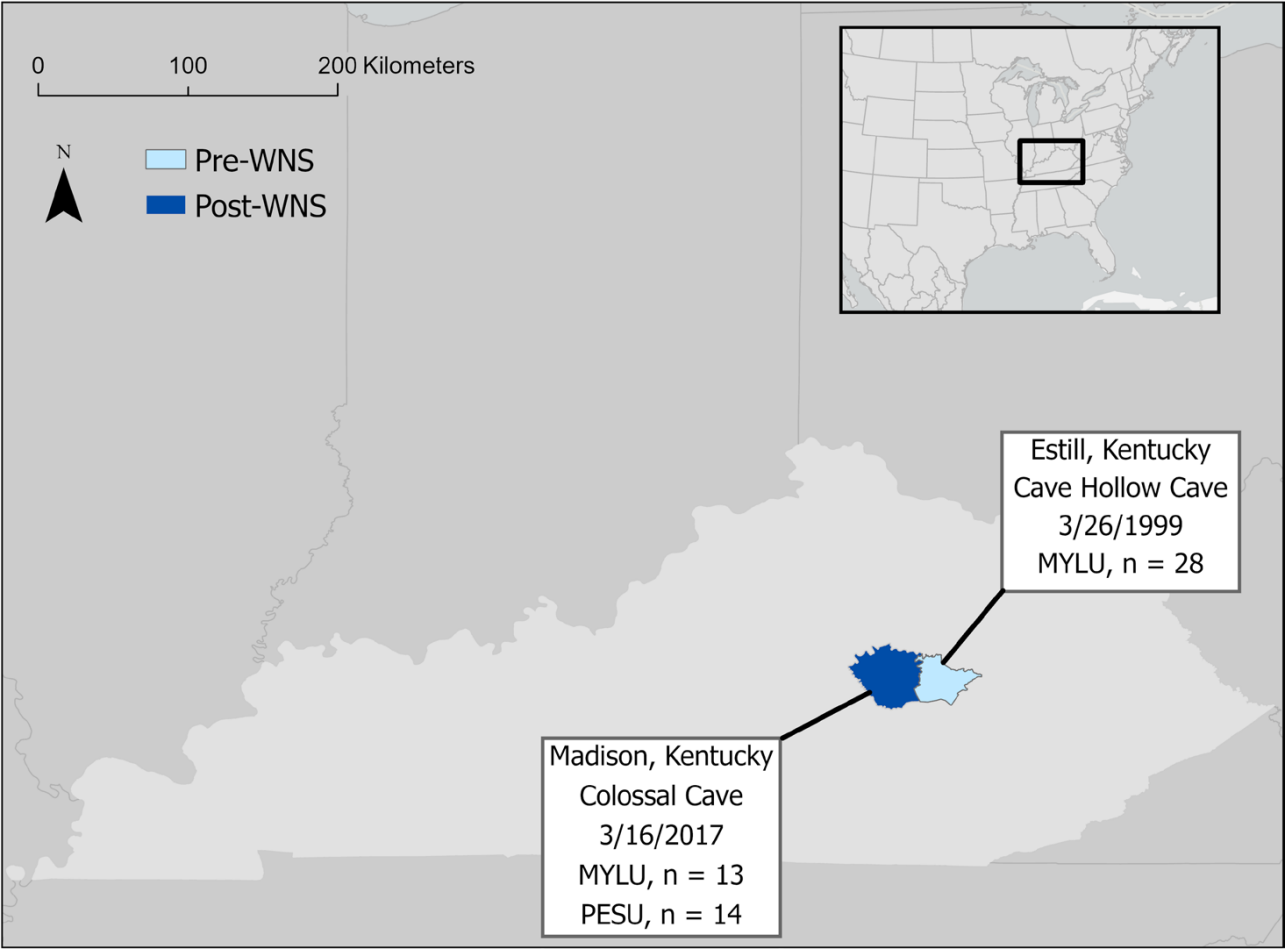
Map of Kentucky showing the counties where pre‐ and post‐WNS samples were collected. Captions show the name of the hibernaculum, date of sample collection, and number of samples collected. Species are demarcated by their 4‐letter acronym, little brown bat = MYLU and tricolored bat = PESU.

We also analyzed 13 little brown and 14 tricolored bat wing biopsy punches from Colossal Cave, Kentucky collected in March 2017. White‐nose syndrome was confirmed at this site in 2013; therefore, these samples represent transcriptional patterns of little brown and tricolored bats that have been exposed to, and were likely infected with, *P. destructans* (hereafter, post‐WNS samples). Indeed, virtually all bats in hibernacula exposed to WNS for more than 2 years become infected (Hoyt et al. 2020). For each sample, we manually removed torpid bats from the hibernaculum substrate and extended one wing against a plastic cutting board. We then used a sterile 2‐mm biopsy punch to collect a tissue sample from a random area of the pelagiopatagium, while avoiding major veins and areas near the edge of the wing that are prone to tearing. Immediately after collection, we transferred tissue samples to sterile 1.5‐mL tubes containing RNALater (Ambion Inc., Austin, Texas). Upon return to Rutgers University, we stored all samples at −80°C until processing. All post‐WNS samples were collected in close coordination with relevant state wildlife agencies under the appropriate permits and approvals (Rutgers IACUC Protocol #: 999900205). Pre‐ and post‐WNS little brown bat samples were used in past work and are further described in Kwait, Eskew, Pinsky, et al. (2025).

### 2.1 Transcriptomic Library Generation

We first cut each wing biopsy sample in half using a scalpel, sterilizing both the scalpel and cutting board between samples. We then extracted total RNA from one half of each sample using the RNeasy micro kit (Qiagen, Hilden, Germany) following the manufacturer's protocol. We constructed mRNA libraries using the Quant‐seq 3′ mRNA‐seq FWD kit (Lexogen, Wien, Austria) according to the manufacturer's instructions. This protocol includes rRNA removal, reverse transcription of RNA into cDNA, and PCR amplification targeting poly‐A tails to capture the 3′ end of mRNA molecules. We quantified resulting prepared libraries using the Qubit dsDNA high sensitivity library quantification kit (Invitrogen, MA, USA) and pooled samples in equimolar ratios into two batches. We submitted samples to the Princeton University Genomics Core for single‐end sequencing on two lanes of an Illumina NovaSeq SP v1.5 with a read length of 100 bp (Kwait et al. 2024).

### 2.2 Bioinformatics

Following sequencing, we demultiplexed raw reads using *BARCODESPLITTER* (Leach and Parsons 2019) and downloaded them to the Amarel high performance computing cluster at Rutgers University. We removed residual rRNA using the *bbtools* package (Bushnell 2014) with the SILVA reference rRNA libraries SSU and LSU (Quast et al. 2012). We then used TRIMMOMATIC to remove low quality beginning and ending bases, reads containing sections with group quality scores < 15, poly‐A tails, adapters, and reads < 36 bp in length (Bolger et al. 2014). We also removed any reads that aligned to human, bacteria, fungi, or virus sequences in GenBank using *FastQScreen* (Wingett and Andrews 2018). We aligned all remaining reads to the reference little brown bat genome (MyoLuc2.0) in ENSEMBL using *STAR aligner* (Dobin et al. 2013), which more accurately aligns transcriptome‐derived data to a genomic reference by accounting for spliced mapping (Dobin et al. 2013).

We tabulated read counts per feature using *HTSeq* (Anders et al. 2015) with the GFF file from the little brown bat genome and combined all samples to generate a read count table. We filtered features to retain those with at least 55 (the number of samples in the study) total reads across all samples and at least 10 reads in a minimum of five samples using the *dplyr* package in RStudio (Chen et al. 2016; Team 2013; Wickham et al. 2015). Finally, we normalized the retained reads using the median of ratios method in the *DESeq2* package (Love et al. 2014).

This dataset has the potential to be influenced by PCR duplication and reference bias because our library preparation included PCR amplification and the alignment of tricolored bat reads to a little brown bat reference genome. We implemented several quality control procedures to evaluate the likelihood that these factors impacted our conclusions. The details and results of these procedures can be found in Appendix B. Overall, we cannot rule out the potential influence of these factors, but we did not find evidence that they strongly impacted our results.

### 2.3 Differential Expression Analysis

Following filtering, we performed differential expression analysis with *DESeq2* (Love et al. 2014), including sample group, sequencing batch, and sex as covariates. We treated sample group as a three‐level categorical variable, considering pre‐WNS little brown bat (“MYLU”) samples as the reference to which post‐WNS MYLU and tricolored bat (“PESU”) samples were compared. To make pairwise comparisons, we also ran the analysis using post‐WNS MYLU as the reference. We considered genes to be significantly differentially expressed if they exhibited a log2‐fold change > 2 and exhibited a false discovery rate (FDR) < 0.05 using the Benjamini‐Hochberg corrected *p*‐value. We identified the biological functions of differentially expressed genes using *GeneCards* (Rebhan et al. 1998).

Inferring the response of tricolored bats from comparison to little brown bat samples is difficult in the absence of unexposed tricolored bat control samples. We considered the relationships between the results of the three differential expression analyses mentioned above to find evidence of the transcriptomic response of tricolored bats to WNS. We regard these findings as preliminary due to the uncertainty inherent in this opportunistic design.

We first identified shared differentially expressed genes among these comparisons, considering a differentially expressed gene to be shared among the groups only if it was regulated in the same direction relative to the reference in both comparisons. This resulted in seven groups of differentially expressed genes, including genes that were shared between each comparison and those that were only differentially expressed in each individual comparison. Of the resulting groups of differentially expressed genes, we considered two interpretable despite analytical uncertainty: (1) differentially expressed genes shared between the post‐WNS MYLU versus pre‐WNS MYLU (hereafter, *little brown bat response*) and the post‐WNS PESU versus pre‐WNS MYLU comparisons but not shared with the post‐WNS PESU versus post‐WNS MYLU comparison and (2) differentially expressed genes that were shared between the post‐WNS PESU versus pre‐WNS MYLU and post‐WNS PESU versus post‐WNS MYLU comparisons but not shared with the little brown bat response.

We consider the first of these two groups as the putative shared response between little brown bats and tricolored bats to WNS. These genes are regulated in the same direction relative to pre‐WNS MYLU in both PESU and MYLU, suggesting the potential for them to be related to the WNS response in both species. It is possible that the expression levels of some of these genes represent baseline levels in PESU despite being differentially expressed relative to pre‐WNS MYLU. However, these genes also are those that do not differ significantly between post‐WNS PESU and post‐WNS MYLU. It therefore appears unlikely that these expression patterns are related to baseline species differences and more likely that they are associated with the infection response.

We consider the second of these two groups as the consistent species differences between tricolored bats and little brown bats. These are the genes that are differentially expressed in the same direction in PESU compared to MYLU regardless of MYLU WNS status. The lack of tricolored bat control makes it unclear if this group of genes would differ between the MYLU groups and pre‐WNS PESU, thus, making it unclear if they relate to the WNS response. Thus, this group of genes is likely composed of species level differences and the genes most subject to reference bias.

We then conducted gene ontology (GO) term analyses on the significantly upregulated and downregulated genes of the putative shared response and the consistent species differences to identify functional terms overrepresented relative to a reference group of functional terms. We performed analyses using the *g:profiler* web platform with the *g:GOSt* function and default parameters, applying an FDR significance threshold < 0.05 (Raudvere et al. 2019). We used all annotated genes within the *Myotis lucifugus* genome as the reference group. Lastly, to broadly compare differential expression between species, we calculated Jaccard similarity indices between differentially expressed genes from the post‐WNS PESU versus pre‐WNS MYLU comparison and the little brown bat response.

### 2.4 Principal Component Regression

We performed a principal components analysis (PCA) on normalized read counts using the *prcomp* function in base R and graphed the first two principal components using *ggbiplot* (Vu 2011). To test for predictors of broad differences in expression, we standardized and centered the values of the first two principal components for each sample, then constructed linear models assuming a normal distribution with each principal component as a continuous response variable and sample group, sex, and sequencing batch as predictors. Sample group again included three categories: pre‐WNS MYLU; post‐WNS MYLU; and post‐WNS PESU. We then calculated Tukey adjusted *p*‐values for pairwise comparisons between the sample group categories from these models using the R package *emmeans* (Lenth et al. 2018). We assessed model fit, dispersion, outlier influence, and residual quantile similarity of the constructed models using the *DHARMa* package in R (Hartig and Hartig 2017). Finally, we extracted the correlations between each gene and the first two principal components to identify which genes contributed strongly to each component. We considered genes with correlation coefficients > 0.5 to be associated with a given principal component, and we determined their functions using *GeneCards* (Rebhan et al. 1998).

### 2.5 Comparison of Relative *Pseudogymnoascus destructans* Load Between Species

We quantified relative *P. destructans* loads among samples by determining the proportion of reads that mapped to the *P. destructans* reference genome. We first filtered reads with *FastQScreen* as described above, this time retaining reads mapping to the fungal genomes and removing sequences aligning to the little brown bat and mouse‐eared bat ( *Myotis myotis* ) reference genomes. We then used *STAR aligner* to map the retained reads to the reference *P. destructans* genome (GCF_001641265.1) in GenBank. To reduce potential nonspecific matches, we excluded any sequences detected in pre‐WNS samples under the assumption that these represented homology with other locally occurring organisms rather than *P. destructans*. We then calculated the relative abundance of *P. destructans* reads, or proportion of the total reads aligning to the *P. destructans* genome. We compared relative *P. destructans* abundance between post‐WNS little brown bats and post‐WNS tricolored bats using a linear model. The model structure matched that used in the principal component regression, with relative *P. destructans* abundance as the response variable and species, sex, and sequencing batch included as covariates.

## 3 Results

In total, we collected 55 samples, including 28 pre‐WNS little brown bats, 13 post‐WNS little brown bats, and 14 post‐WNS tricolored bats (Table A1). After initial read filtering, we obtained sufficient sequencing data for 53 samples: 26 pre‐WNS little brown bats, 13 post‐WNS little brown bats, and 14 post‐WNS tricolored bats. After final filtering, samples retained an average of 253,675 ± 11,505 (±SEM) reads, mapped to a total of 5440 genes.

Quality control analyses (Appendix B) suggested that samples were comparable between groups. However, tricolored bat samples had higher mismatch rates and lower mapped lengths than either group of little brown bat samples. Analyses of reference bias did not indicate an obvious impact on our results, including a rerun of the analyses with a more lenient mismatch threshold. Duplication rates were high in this dataset but followed a pattern consistent with true biological variation. The series of analyses we ran did not signal a strong impact of duplication on our results, including a rerun of the differential expression and PC regression excluding samples with more problematic duplication profiles. While we cannot rule out the influence of reference bias or PCR duplication on our results, these analyses did not uncover evidence of substantial impacts.

The analysis of post‐WNS PESU versus pre‐WNS MYLU identified 983 significantly differentially expressed genes, including 636 upregulated and 347 downregulated genes (Table S1). The analysis of the little brown bat response identified 351 differentially expressed genes, of which 203 were upregulated and 148 were downregulated. The analysis of post‐WNS PESU versus post‐WNS MYLU identified 709 genes consisting of 441 upregulated and 268 downregulated genes. Jaccard similarity between the post‐WNS PESU versus pre‐WNS MYLU differentially expressed genes and those of the little brown bat response was 0.11.

After comparing the three lists of differentially expressed genes, the putative shared response to WNS consisted of 122 genes, including 71 upregulated and 51 downregulated genes (Figure 2). The putative shared response included multiple genes associated with immune functions, including C1R, CUEDC2, PGLYRP1, and CSRP2 (Table 1A). Other genes in the response were associated with metabolic function and circadian rhythms including MIOS and NR1D1 (Table 1A, Table S2A). There were no over‐represented GO‐terms among upregulated genes of the putative shared response. Three GO‐terms were over‐represented among downregulated genes of the shared response, which included lactic acidosis, acidosis, and abnormality of pH homeostasis (Table 1B). Despite the large difference in gene identity between the little brown bat response and the post‐WNS PESU versus pre‐WNS MYLU comparison, many genes in the same functional category were differentially expressed in both analyses (Table 2). For example, major histocompatibility complex classes I or II was only functionally associated with two genes in the putative shared response but was functionally associated with 11 additional genes that were differentially expressed in the post‐WNS PESU versus pre‐WNS MYLU analysis only and five genes in the little brown bat response only (Table 2).

**FIGURE 2:**
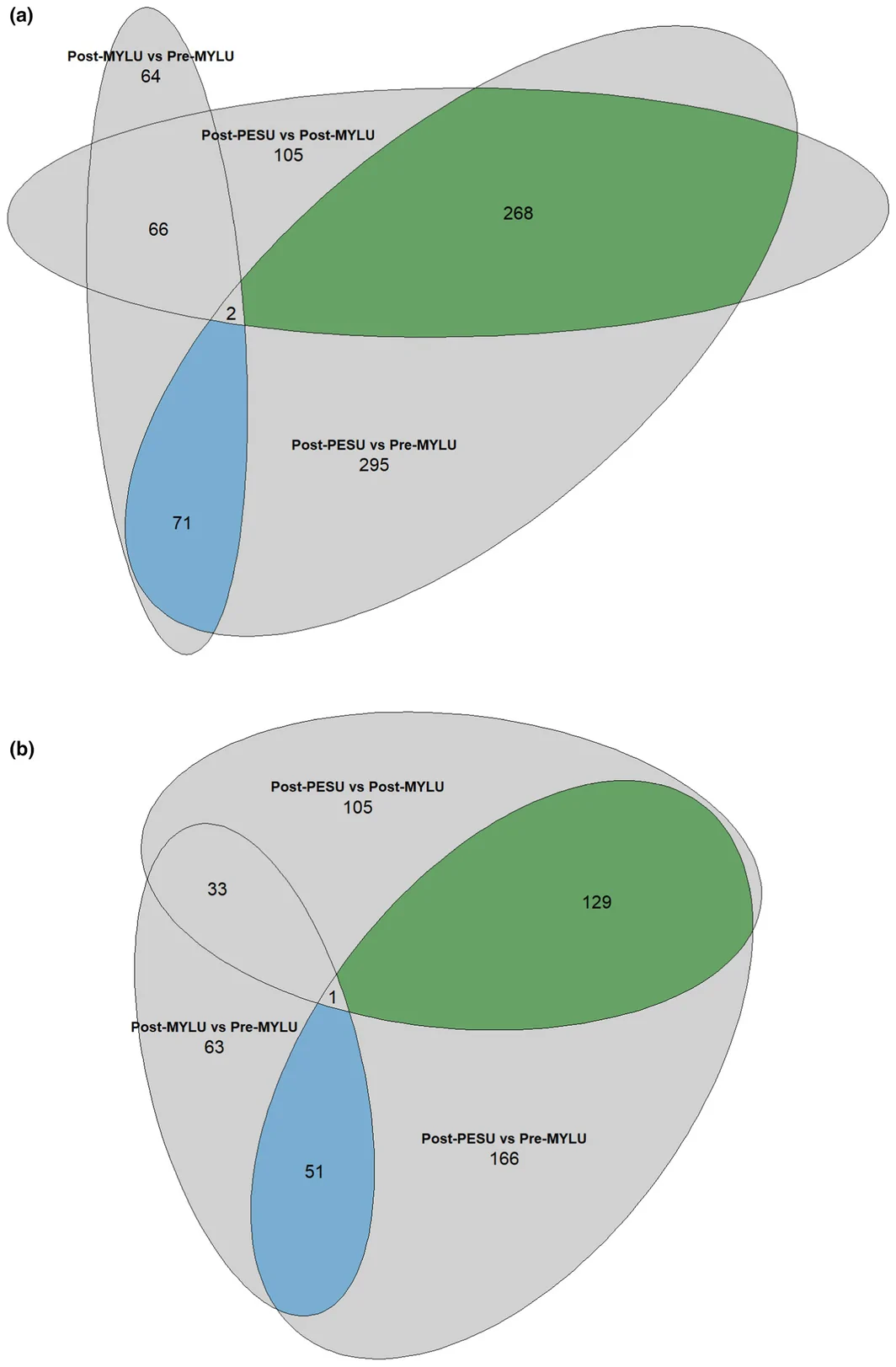
Venn diagram comparing the significantly differentially expressed genes between each pair of differential expression analyses. Panel (a) consists of the genes that were upregulated in each comparison. Panel (b) contains the genes that were downregulated. The highlighted regions represent the two areas of focus in this study. We consider the overlap between the post‐WNS MYLU versus pre‐WNS MYLU and post‐WNS PESU versus pre‐WNS MYLU comparisons as the putative shared response to WNS (shown in blue). We are considering the overlap between the post‐WNS PESU versus pre‐WNS MYLU and post‐WNS PESU versus post‐WNS MYLU comparisons as consistent species‐level differences in gene expression (shown in green).

**TABLE 1:** (A) Truncated list of genes that were differentially expressed in the putative shared response (in both the post‐PESU vs. pre‐MYLU and post‐MYLU vs. pre‐MYLU comparisons). The Effect for each species (P for PESU and M for MYLU) refers to the log2‐fold change, and the FDR for each species refers to the Benjamini‐Hochberg corrected *p*‐value. The Function is a summarized version of the description of each gene from *GeneCards* (Rebhan et al. 1998). Full results from *DESeq2* available in Table S2A. (B) List of overrepresented GO terms among differentially expressed genes in the putative shared response. Regulation shows whether the term was overrepresented among upregulated or downregulated genes. Full GO term results are available in Table S2B.

(A)					
Gene	Effect (P)	FDR (P)	Effect (M)	FDR (M)	Function
PGLYRP1	2.34	0.017	4.50	0.0004	Innate immune response, defensin, apoptosis, lymphocyte activation, antimicrobial
TXNDC5	3.15	1.06 × 10^−5^	3.36	0.0003	Protein folding, hypoxia, apoptosis, innate immune system
CUEDC2	2.45	0.0046	3.11	0.012	Cytokine production, inflammation, macrophage, TLR signaling
CSRP2	−2.33	0.0056	−2.73	0.016	Immune response, downregulated in response to cell injury
NR1D1	−3.04	1.99 × 10^−8^	−2.40	0.001	Circadian rhythms and metabolic pathways, inflammation, NF‐KB, gluconeogenesis, adipogenesis
C1R	−2.17	2.84 × 10^−6^	−2.07	0.0008	Subunit of complement system, innate immune system, phagocytosis, inflammation
LDHB	−3.72	0.002	−4.28	1.05 × 10^−6^	Glycolysis, metabolic states and circadian oscillators
ENO1	2.62	0.0005	2.62	0.021	Glycolysis, gluconeogenesis, many functions including growth control, hypoxia tolerance, and allergic reactions
TEC	4.18	4.37 × 10^−10^	2.91	0.0043	Cytokine receptors, lymphocyte surface antigens, key player in regulation of immune functions, signaling in T‐cell helper, cytokine production in mast cells

(B)		
GO term	Regulation	FDR
Lactic acidosis	Down	0.0008
Acidosis	Down	0.0062
Abnormality of pH homeostasis	Down	0.0101

**TABLE 2:** List of significantly differentially expressed genes that were unique to the analysis of each species and shared between them in comparison to pre‐WNS MYLU for a group of functional categories.

Functional Term	Unique to tricolored bats	Shared	Unique to little brown bats
Cytokines	CD47	PHB2	OSMR
	TNFSF10	MAPKAPK3	MKNK2
	ATL1	CUEDC2	PLK2
	IRAK3	GNB1	
	NMI	TEC	
	ANXA1		
	ATG16L1		
	HCST		
	F3		
	GNAI3		
	RASGRP1		
	SOCS6		
	GPNMB		
	TGFBR2		
	OTUB1		
	WDFY1		
MHC	UBE2L3	PSMA3	SPSB3
	KEAP1	PSMC6	HIVEP1
	YJEFN3		XBP1
	PSMB9		RNF19B
	GNL1		HERC4
	RNF126		
	RNF217		
	UBE2J2		
	PSMB1		
	LRR1		
	RNF114		
Inflammation	F3	PHB2	GAPDH
	RARRES2	MAPKAPK3	EREG
	DCN	PARP12	HPSE
	DMKN	CUEDC2	TRAF3
	CLYD	GNB1	
		C1R	
		NR1D1	
		DDIT3	
Starvation response or ATG	TMEM59	MIOS	SNX7
	ATG4D		SUPT20H
	GPR155		ATG2A
	FOXK2		FNIP1
	ATG101		EMC6
	ATG16L1		RAB7A

*Note:* Genes were included in a functional category if the functional term was contained within the *GeneCards* summary.

The consistent species difference group consisted of 397 genes, of which 268 were upregulated and 129 were downregulated relative to the MYLU samples in both analyses. Differentially expressed genes had a variety of functions with some relating to immune activity, including IRAK3, TNFSF10, CYLD, and F3 (Table 3, Table S3A). Others related to metabolic function, such as CROT and ACSM3. There were 22 over‐represented GO‐terms among upregulated genes and eight among downregulated genes (Table S3B). The over‐represented GO‐terms almost entirely related to general cellular structures and processes such as cytoplasm, translation, and structural molecule activity. Such processes are difficult to directly link to the WNS response.

**TABLE 3:** Examples of genes with functions related to immune and metabolic systems that were consistently differentially expressed between species (in both the post‐WNS PESU vs. pre‐WNS MYLU and the post‐WNS PESU vs. post‐WNS MYLU comparisons).

Gene	Function
IRAK3	TLR signaling regulation, IL‐1 signaling, innate immune response, NF‐kB signaling, cytokine signaling
TNFSF10	This is a cytokine, apoptosis, glucocorticoid regulation, TGF‐Beta pathway
CYLD	Deubiquitinase activity, NF‐kB regulation, innate immune response, inflammation, necroptosis, microtubule regulation
F3	Blood coagulation, inflammation, upregulated in response to inflammatory stimuli, enables cytokine receptor activity, cellular immune response
CROT	Lipid metabolism, fatty acid beta‐oxidation
ACSM3	Fatty acid coa ligase activity, acyl‐coa metabolic process, fatty acid biosynthetic process, hypertension, mitochondrial fatty acid beta‐oxidation

*Note:* Functions are truncated summaries of the *GeneCards* descriptions. Full results can be found in Table S3A.

### 3.1 Principal Component Regression

In the PCA analysis, PC1 explained 76.7% of the variance in the dataset, and PC2 explained 7.4%. The PCA biplot suggested high variation of PC1 in post‐WNS little brown bats, which extended far from the other two, more clustered groups (Figure 3). There was separation among all three groups along PC2 (Figure 3). For both principal components, sample group was the only meaningful explanatory covariate, while sex and sequencing batch showed no significant effects (Table C1). Additional modeling quality control steps are outlined in Appendix C.

**FIGURE 3:**
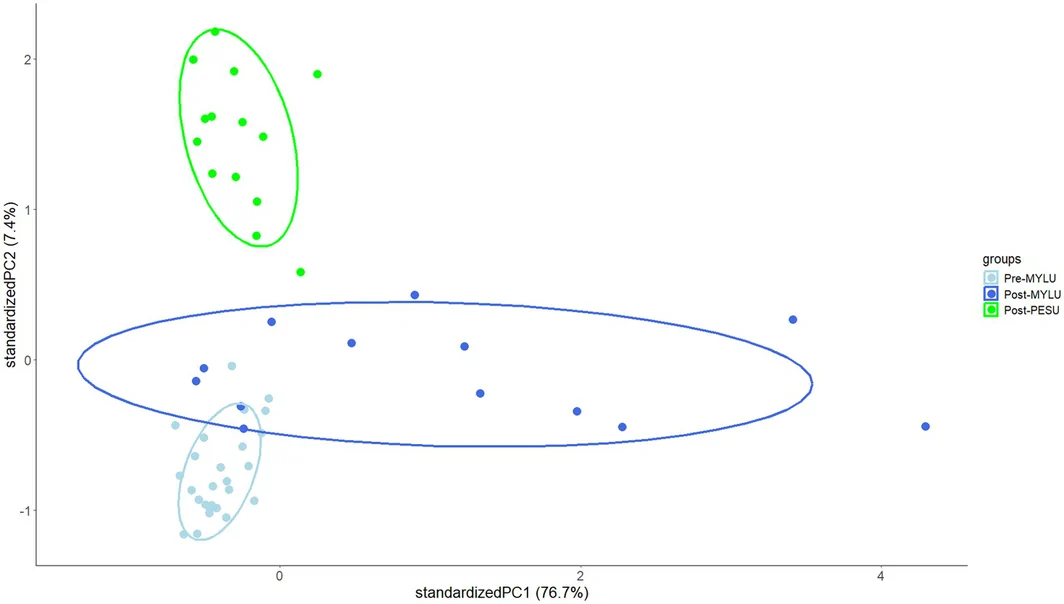
PCA biplot of normalized read counts for each sample group. Groups are encircled with one standard deviation ellipses.

Pairwise comparisons demonstrated significantly higher PC1 values in post‐WNS little brown bats relative to both pre‐WNS little brown bats and post‐WNS tricolored bats (Table 4). PC1 values did not differ significantly between pre‐WNS little brown bats and post‐WNS tricolored bats. Pairwise comparisons of PC2 values revealed significant differences among all three groups, with the highest values observed in post‐WNS tricolored bats, followed by post‐WNS little brown bats (Table 4). PC1 was strongly correlated with expression of two genes: S100A12 (*r* = 0.696) and S100A8 (*r* = 0.657). The next highest correlation was with S100A7 (*r* = 0.249). PC2 was highly correlated with S100A4 (*r* = 0.541), followed by Spink5 (r = 0.378). Expression patterns of genes with correlations > 0.5 corresponded closely with the distribution of principal component values among sample groups (Figure 4).

**TABLE 4:** Model results for pairwise comparisons of sample group by principal components.

Model set	Query	Reference	Estimate	*p*
PC1	Post‐MYLU	Post‐PESU	1.353 [0.694, 2.012]	0.0005
PC1	Post‐MYLU	Pre‐MYLU	1.469 [0.853, 2.085]	0.0001
PC1	Post‐PESU	Pre‐MYLU	0.116 [−0.411, 0.643]	0.901
PC2	Post‐MYLU	Post‐PESU	−1.660 [−1.951, −1.369]	< 0.0001
PC2	Post‐MYLU	Pre‐MYLU	0.568 [0.297, 0.839]	0.0004
PC2	Post‐PESU	Pre‐MYLU	2.228 [1.996, 2.460]	< 0.0001

*Note:* Model set demonstrates if the results are from the model including PC1 or PC2 as a response variable. Query shows which group is being tested and reference shows which group is being used as a reference. Estimate refers to the model derived coefficient with 95% confidence intervals. The *p*‐value is the Tukey adjusted *p*‐value for pairwise comparisons.

**FIGURE 4:**
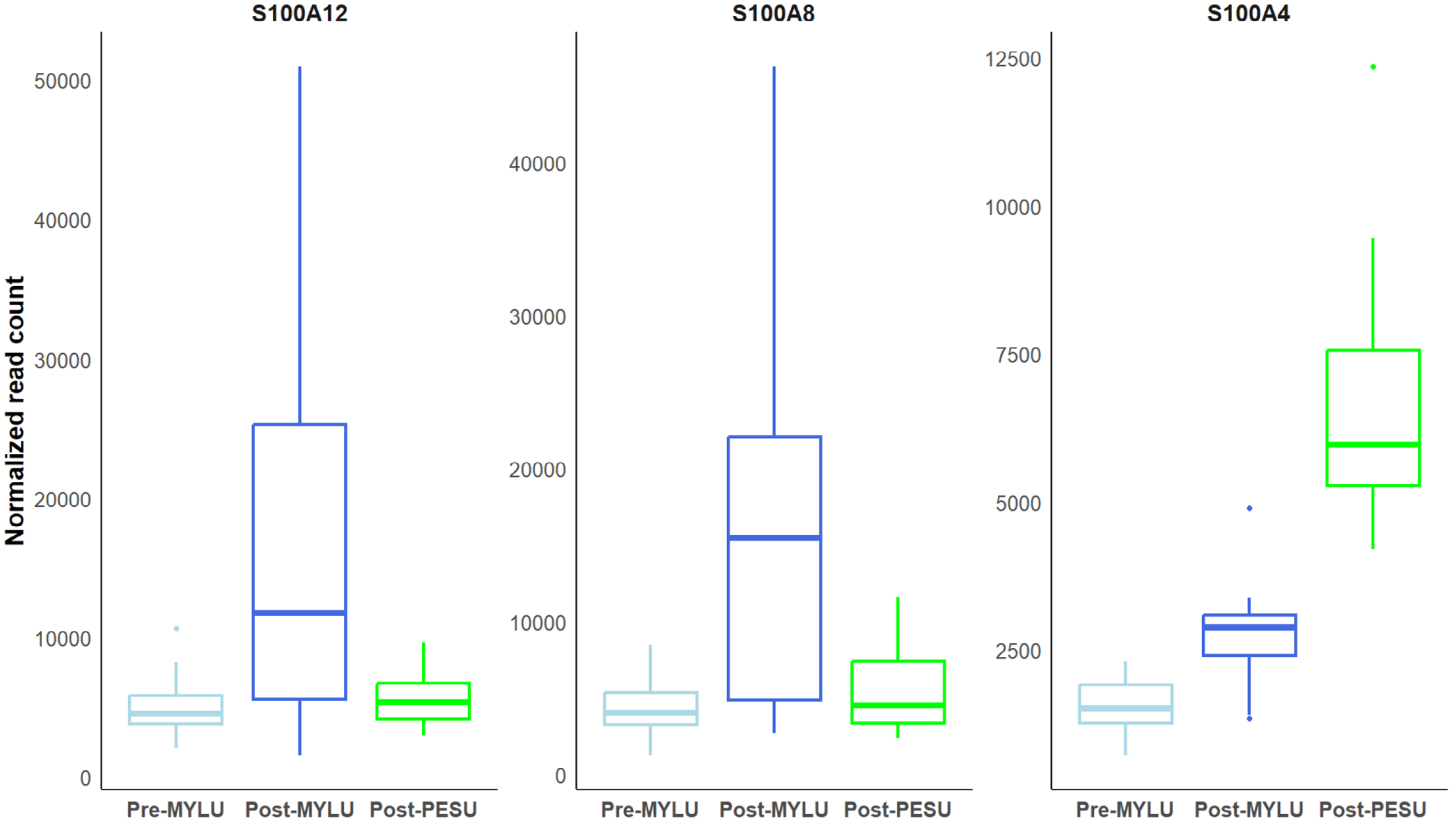
Boxplots comparing the normalized read counts of the genes most correlated with the first two principal components between the sample groups of pre‐WNS little brown bats, post‐WNS little brown bats, and post‐WNS tricolored bats. The midline denotes the median, the hinges show the first and third quartiles, and the whiskers extend from the first and third quartiles to 1.5× the interquartile range.

### 3.2 Comparison of Relative *Pseudogymnoascus destructans* Load Between Species

After filtering, post‐WNS little brown bat and tricolored bat samples contained an average of 636 ± 175 reads aligning to the *P. destructans* genome, representing 1.88 × 10^−4^% ± 4.05 × 10^−5^% of total reads. Tricolored bats had a significantly lower proportion of reads aligning to *P. destructans* than little brown bats (Table D1). Details of statistical results can be found in Appendix D.

## 4 Discussion

White‐nose syndrome has caused severe population declines in several North American bat species, yet the molecular mechanisms underlying species‐specific disease responses remain incompletely understood. Our results suggest that tricolored bats likely mount a pronounced transcriptomic immune response to *P. destructans* infection that broadly resembles the response previously described in little brown bats. Without pre‐WNS tricolored bat samples, it is difficult to determine what portion of the observed differential expression relates to the WNS response of tricolored bats versus constitutive differences in expression between tricolored bats and little brown bats. However, the similarities between the differential expression patterns observed in the putative shared response (tricolored bats and little brown bats) and previous work on little brown bats support the hypothesis that tricolored bats exhibit a pronounced transcriptomic response that is maintained through the hibernation season.

Immune system elements implicated in the putative shared response include inflammation, the complement cascade, Toll‐like receptor signaling pathways, immune‐associated transcription factors such as NF‐kb, and pathways involving cytokine signaling and immune cell activation (e.g., macrophages and lymphocytes). For example, we observed upregulation of one gene associated with the complement cascade (C1R), which is an integral part of the initiation of immune and inflammatory responses (Almitairi et al. 2018; Heesterbeek et al. 2018; Ricklin et al. 2010). In addition, we observed upregulation of several genes associated with the Toll‐like receptor 7/8 signaling pathway, including MAPKAPK3, RPS6KA1, and CUEDC2 (Rebhan et al. 1998). Toll‐like receptors 7 and 8 are transmembrane receptors that recognize pathogens and initiate an innate immune response through activation of transcription factors such as NF‐kb, ultimately promoting the production of proinflammatory cytokines (Li et al. 2023). Notably, activation of these pathways has also been reported in previous transcriptomic studies of little brown bats with WNS (Davy et al. 2020; Field et al. 2018; Kwait, Eskew, Pinsky, et al. 2025; Lilley et al. 2019; Moore et al. 2011). Past WNS transcriptomic studies have targeted immune responses during interbout arousal, holding individuals for 60 min prior to sampling and has found that the majority of the immune response occurs while bats are normothermic (Field et al. 2018). Our sampling was performed soon after bats were removed from the hibernaculum substrate and, thus, likely at early stages of arousal. The exact impact of this difference is unclear, but the patterns observed here may relate to the activation of the immune response during early stages of arousal.

Some transcriptomic activity we observed in the putative shared response may relate to pathological immune dysregulation. For example, the GO terms *lactic acidosis*, *acidosis*, and *abnormality of acid–base homeostasis* were overrepresented among differentially expressed genes shared between tricolored bat and little brown bat responses. Acidosis refers to a decrease in blood pH caused by metabolic or respiratory disruption (Lim 2007; Patel and Sharma 2023). Such disruption can arise from CO_2_ accumulation or electrolyte imbalance (Kregenow et al. 2006; Moini and Ferdowsi 2024), both of which have been described in bats with WNS (Cryan et al. 2013; Verant et al. 2014). Lactic acidosis occurs when acidification is caused by excess lactate (Suetrong and Walley 2016), and one of its most common causes is septic shock (Jansen et al. 2010; Suetrong and Walley 2016). Supporting the possibility of immune dysregulation, the gene PGLYRP1 was also upregulated in tricolored bats. PGLYRP1 responds to pathogen antigens and has recently been identified among a core set of genes associated with sepsis diagnosis (Chen et al. 2025; Royet and Dziarski 2007). Potential indicators of systemic inflammation and possible sepsis have also previously been observed in little brown bats with WNS (Kwait, Eskew, Pinsky, et al. 2025; Meteyer et al. 2012).

However, the interpretation of these transcriptomic signals remains uncertain. For example, lactic acidosis can occur naturally in hibernating mammals as a consequence of hypovolemia associated with torpor (Malan 1980, 2014; Milsom and Jackson 2011), and although sepsis is the most common cause, several other physiological disturbances can lead to lactic acidosis (Schuh et al. 2018). Consequently, the relationship between these transcriptional signals and WNS pathophysiology requires further investigation.

In addition to immune‐related pathways, tricolored bats exhibited evidence of metabolic and circadian changes. Several genes related to aspects of metabolism were differentially expressed in the putative shared response. For example, MIOS, which controls autophagy regulation during amino acid starvation (Rebhan et al. 1998; Shinhmar et al. 2024), was downregulated in both species compared to pre‐WNS little brown bats. Such transcriptional changes may reflect nutritional stress associated with WNS, which increases energy expenditures through increased torpor arousals and depletion of fat reserves (Reeder et al. 2012; Verant et al. 2014). Four genes with functions related to circadian rhythms (NR1D1, LDHB, NFIL3, DNM1L) (Rebhan et al. 1998) were differentially expressed in the putative shared response and, thus, are likely related to the response of tricolored bats to WNS. Circadian mechanisms have been hypothesized to influence hibernation timing and arousal patterns (Kwait et al. 2024; Malan 2010; Willis and Wilcox 2014), suggesting that differential expression of these genes may relate to the disruption of torpor cycles observed in bats with WNS or differences in torpor patterns between species.

Overall, the apparent transcriptomic response of tricolored bats to WNS mirrors much of the response previously documented in little brown bats. Both species show activation of immune pathways, metabolic changes, and transcriptional signals consistent with prolonged physiological stress (Lilley et al. 2017; Moore et al. 2011, 2013; Whiting‐Fawcett et al. 2021). Because *P. destructans* infection typically occurs early in the hibernation season (Langwig et al. 2015), and these samples were collected in March, the continued immune activation suggests that this response may persist throughout most of the hibernation period. Such prolonged immune activity is characteristic of susceptible host responses in many disease systems, where gene expression changes persist long after initial infection (Eskew et al. 2021). Similar patterns have been reported in little brown bats (Kwait, Eskew, Gignoux‐Wolfsohn, et al. 2025; Lilley et al. 2019). In contrast, species considered less susceptible to WNS, such as big brown bats ( *Eptesicus fuscus* ) and greater mouse‐eared bats ( *Myotis myotis* ), do not display prolonged immune activation following pathogen exposure (Davy et al. 2020; Lilley et al. 2019; Whiting‐Fawcett et al. 2024).

Despite these broad functional similarities, many differentially expressed genes were unique to each species. For example, separate dyads of genes were associated with the starvation response in each species: in tricolored bats these included FOXK2 and GPR155, whereas in little brown bats they were SUPT20H and SNX7 (Rebhan et al. 1998). Only one gene associated with the response to cellular starvation, MIOS, was present in the putative shared response. In addition, post‐WNS little brown bats demonstrated increased expression of S100A12 and S100A8 compared to unexposed individuals. These genes drove the largest axis of variation in the PCA and have been repeatedly identified in previous studies of the little brown bat response to WNS (Field et al. 2015, 2018; Lilley et al. 2019), suggesting they represent an important component of that pathway. Both genes regulate immune activity, and their overexpression has been associated with systemic inflammation and sepsis in other taxa (Dubois et al. 2019; Guo et al. 2021; Payen et al. 2008; Sehgal et al. 2022).

In contrast, tricolored bats did not show increased expression of these genes relative to pre‐WNS little brown bats. Instead, tricolored bats exhibited elevated expression of S100A4 relative to both pre‐ and post‐WNS little brown bats. S100A4 belongs to the same protein family (S100) and participates in several immune functions, including cytokine signaling and inflammatory regulation (Ambartsumian et al. 2019; Wong et al. 2025). S100A4 has also been associated with chronic inflammatory conditions and altered immune function during prolonged infection (Wherry et al. 2007; Wong et al. 2025). These results suggest that although the overall immune response to WNS likely involves similar transcriptional pathways and shares some of the same genes, many of the specific genes driving those responses differ between species. Similar patterns have been observed among isolated populations of species, with rapid adaptation acting on different genes within the same functional groups. For example, populations of wild killifish demonstrated parallel rapid evolution in response to toxicity, but selection did not act on the same genes within each population (Reid et al. 2016). Thus, differences in gene expression between tricolored and little brown bats could relate to parallel immune adaptations that evolved under the same evolutionary pressure and therefore have similar functional consequences even though they involve different suites of genes.

The implications of these differences for WNS pathophysiology remain unclear. It is possible that similar functional outcomes arise through different molecular pathways in each species or that these transcriptional differences reflect meaningful variation in disease response. The relationship of these gene expression patterns to WNS is further obscured by the fact that we compared post‐WNS tricolored bats to pre‐WNS little brown bats, and thus, we cannot be sure whether the observed changes in gene expression are a response to infection or if they represent baseline differences between species. Comparing transcription patterns between tricolored bats pre‐ and post‐WNS would allow us to directly interrogate the WNS response in this species. Further comparative studies of WNS pathophysiology across species can help clarify the relative contributions of constitutive gene expression and gene expression associated with the immune response.

At the time the post‐WNS samples used in this study were collected, WNS had been present in North America for approximately a decade, and evidence suggests that rapid evolutionary responses had already begun in some bat populations (Gignoux‐Wolfsohn et al. 2021; Kwait et al. 2024). Several little brown bat populations have shown signs of recovery following initial disease‐induced declines (Hoyt et al. 2021; Maslo et al. 2015; Reichard et al. 2014). Physiological adaptations and genomic signatures of selection (Auteri and Knowles 2020; Cheng et al. 2019; Donaldson et al. 2017; Frank et al. 2019; Gignoux‐Wolfsohn et al. 2021; Lilley et al. 2016) suggest that WNS has driven evolution from standing genetic variation. However, not all species appear to be adapting to WNS (Kwait, Eskew, Gignoux‐Wolfsohn, et al. 2025), and it remains unclear whether tricolored bats are undergoing similar evolutionary responses. Some tricolored bat populations have shown increased growth rates following initial declines, and some individuals continue to survive despite repeated annual infections (Hoyt et al. 2021).

Given this uncertainty, it is unclear if the transcriptomic responses we observed would occur in naïve bats during initial exposure or if the signal also reflects adaptation or acclimation to WNS. Little brown bats from populations that have been exposed to WNS across multiple generations appear to maintain an aggressive immune response thought to be indicative of susceptibility, but the associated gene expression is altered in comparison to little brown bats during first exposure (Lilley et al. 2019; Moore et al. 2013). In addition, some of the loci under selection due to WNS impact the expression of multiple immune genes, demonstrating an evolutionary impact on transcription patterns (Kwait et al. 2024). Together, these past studies suggest the gene expression patterns in post‐WNS little brown bats that have persisted with WNS for multiple years are likely different from those of naïve individuals infected for the first time. The degree to which tricolored bats have adapted or acclimated to WNS is currently unknown but could similarly impact transcription patterns.

Little brown bats had a significantly higher proportion of reads aligning to the *P. destructans* genome than tricolored bats. This difference has the potential to relate to the variation in immune gene expression between species observed here. However, this question cannot be answered with confidence based on the data in this study. In addition, the amount of fungal RNA found in this study was quite low compared to previous work, likely related to the random sampling design employed in comparison to designs targeting lesions. A meta‐analysis of fungal loads in North American bats found a similar trend between these species, with slightly higher loads typically in little brown bats (Hoyt et al. 2021).

Genes that were differentially expressed in tricolored bats relative to little brown bats both before and after WNS may reflect baseline differences between the species. Functions of this group of genes were widely variable, including lipid metabolism, skin integrity, and cell shape. GO‐term analysis suggested general cellular functions and structures such as structural molecule activity, protein biosynthetic process, and organelles were overrepresented. The physiological consequences of these transcriptional differences remain uncertain.

## 5 Limitations

Several aspects of experimental design should be considered when interpreting these results. First, the tricolored bat response to WNS was inferred through comparison with pre‐ and post‐WNS little brown bats. As discussed throughout the manuscript, apparent differences in gene expression could therefore reflect species‐level differences unrelated to infection. Overlap in immune pathways between species that were regulated in the same direction relative to pre‐WNS MYLU (putative shared response) supports the relevance of at least a portion of the detected transcriptional responses to WNS pathology. The differential expression between post‐WNS PESU and pre‐WNS MYLU that was not shared with the little brown bat response may largely represent constitutive differences between species. Supporting this, the number of differentially expressed genes in this category far outweighed both the little brown bat and putative shared responses. However, these differences, even if constitutive, could still impact WNS pathology. Immune genes within this category, especially, may impact the mechanism by which tricolored bats react to infection.

We also aligned tricolored bat reads to a reference little brown bat genome, suggesting some differences in expression could relate to reference bias. Also, the pre‐WNS samples were collected from a different hibernaculum ~20 years earlier and stored in different media than the post‐WNS samples. However, our alignment quality metrics were broadly comparable between species, and analyses of reference bias did not indicate an obvious influence on results (Appendix B). Additionally, the sampled sites are geographically close, and population genetic studies indicate high gene flow at this scale in both little brown bats (Gignoux‐Wolfsohn et al. 2021; Kwait et al. 2024; Miller‐Butterworth et al. 2014; Vonhof et al. 2015) and tricolored bats (Henderson 2025; Martin et al. 2022), implying that population‐level differences are unlikely to explain the observed expression patterns.

Duplication rates were high in this dataset, and we did not use unique molecular identifiers (UMI) during library preparation. Thus, it is possible that the results presented here are influenced by technical artifacts rather than biological signals. UMIs are the most reliable method of dealing with duplicates in RNA‐seq data, but, in their absence, it is typically recommended to avoid removing duplicates because they cannot be isolated from true biological variation in gene expression (Klepikova et al. 2017). We ran a series of analyses that did not indicate any obvious problems associated with duplication, including a rerun of the analysis excluding samples with the most problematic duplication profiles (Appendix B).

Finally, we did not guide the collection of wing biopsies with UV light to target areas of the wing with damage caused by *P. destructans*, as has been done in several recent transcriptomic studies (Davy et al. 2020; Lilley et al. 2019). Consequently, our sampling design does not distinguish between local and systemic responses. However, this approach allowed the inclusion of previously collected samples and enabled analysis of transcriptomic responses in a larger number of wild individuals. In addition, we detected *P. destructans* RNA in every post‐WNS sample, suggesting that sampled individuals were infected. Despite presence in every sample, the proportion of reads attributed to *P. destructans* was very low, suggesting sampling did not capture large lesions. Because nearly all bats in hibernacula harboring the pathogen for multiple years become infected (Hoyt et al. 2020), it is likely that the transcriptional signals observed here reflect, at least in part, WNS infection.

Future work including an uninfected control tricolored bat group, UV guidance of sample collection to separate systemic and local responses, and alignment to a tricolored bat reference genome would be useful in more explicitly targeting the tricolored bat response to WNS.

## 6 Conclusion

Our results suggest that tricolored bats mount an aggressive transcriptomic response to *P. destructans* infection that includes activation of innate immune pathways, inflammatory signaling, and metabolic processes associated with disrupted hibernation physiology. These responses broadly resemble those previously described in little brown bats, suggesting that both species exhibit immune activation patterns typical of susceptible hosts in wildlife disease systems. Despite these mechanistic similarities, the suite of genes driving the responses was not all identical between species, indicating that comparable functional outcomes may arise through distinct molecular mechanisms. Understanding how these differences influence WNS pathophysiology and survival will require further investigation, particularly as bat populations continue to experience disease‐induced ecological and evolutionary pressures. We report these results as preliminary given limitations associated with the design of the between species comparisons. Continued comparative studies of transcriptomic and physiological responses across bat species can help clarify what drives differences in survival in populations threatened by disease.

## Author Contributions

**Robert E. Kwait:** conceptualization (lead), data curation (lead), formal analysis (lead), visualization (lead), writing – original draft (lead). **Evan A. Eskew:** conceptualization (equal), formal analysis (equal), methodology (supporting), validation (supporting), visualization (supporting), writing – review and editing (equal). **Maanasvi Balasubramanian:** data curation (supporting), formal analysis (supporting), writing – review and editing (supporting). **Sarah A. Gignoux‐Wolfsohn:** formal analysis (supporting), writing – review and editing (supporting). **Malin L. Pinsky:** formal analysis (supporting), writing – review and editing (supporting). **Brooke Maslo:** conceptualization (supporting), formal analysis (supporting), funding acquisition (lead), project administration (lead), writing – review and editing (lead).

## Funding

This work was supported by Rutgers, The State University of New Jersey, Eppley Foundation for Research, and United States Fish and Wildlife Service (FP19AP00595).

## Conflicts of Interest

The authors declare no conflicts of interest.

## Supporting information


**Table S1:** Differential expression analysis results for each comparison. The comparisons are denoted in the first column. The denoted functions are truncated descriptions from GeneCards.
**Table S2:** Differentially expressed genes and GO‐term analysis results for the putative shared response. (A) Genes that were differentially expressed in the same direction in both the post‐WNS PESU vs. pre‐WNS MYLU and post‐WNS MYLU vs. pre‐WNS MYLU comparisons but not in the post‐WNS PESU vs. post‐WNS MYLU comparison. The regulation column shows whether each gene was upregulated or downregulated relative to pre‐WNS MYLU in both analyses. (B) GO term analysis results for the putative shared response as output by g:profiler. The regulation column shows whether the GO term was overrepresented among upregulated or downregulated genes.
**Table S3:** Differentially expressed genes and GO‐term analysis results for the consistent species differences. (A) Genes that were differentially expressed in the same direction in both the post‐WNS PESU vs. pre‐WNS MYLU and post‐WNS PESU vs. post‐WNS MYLU comparisons but not in the post‐WNS MYLU vs. pre‐WNS MYLU comparison. The regulation column shows whether each gene was upregulated or downregulated relative to the MYLU sample group in both analyses. (B) GO term analysis results for the consistent species differences as output by g:profiler. The regulation column shows whether the GO term was overrepresented among upregulated or downregulated genes.

## Figures and Tables

**FIGURE B1:**
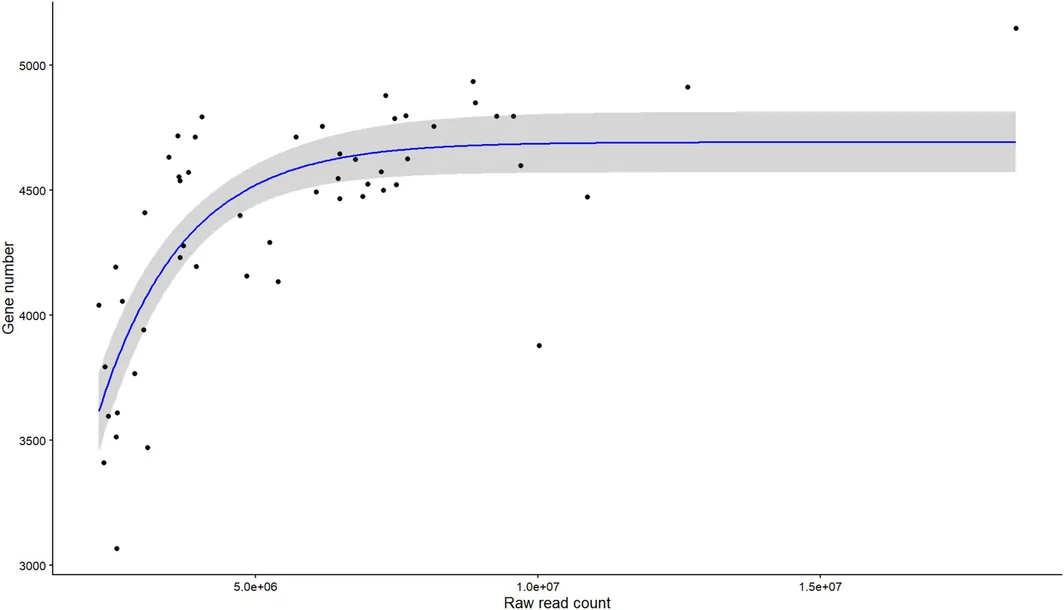
The relationship between the number of discovered genes with a count > 0 and the total raw read count. The trend line represents the slope of the relationship with 95% confidence intervals fit with a negative exponential function. Model summary: *A* = 4963, [95% CI: 4571, 4820] genes, *p* < 0.0001; *k* = 6.62 × 10^−7^ [95% CI: 5.82 × 10^−7^, 7.64 × 10^−7^], *p* < 0.0001.

**FIGURE B2:**
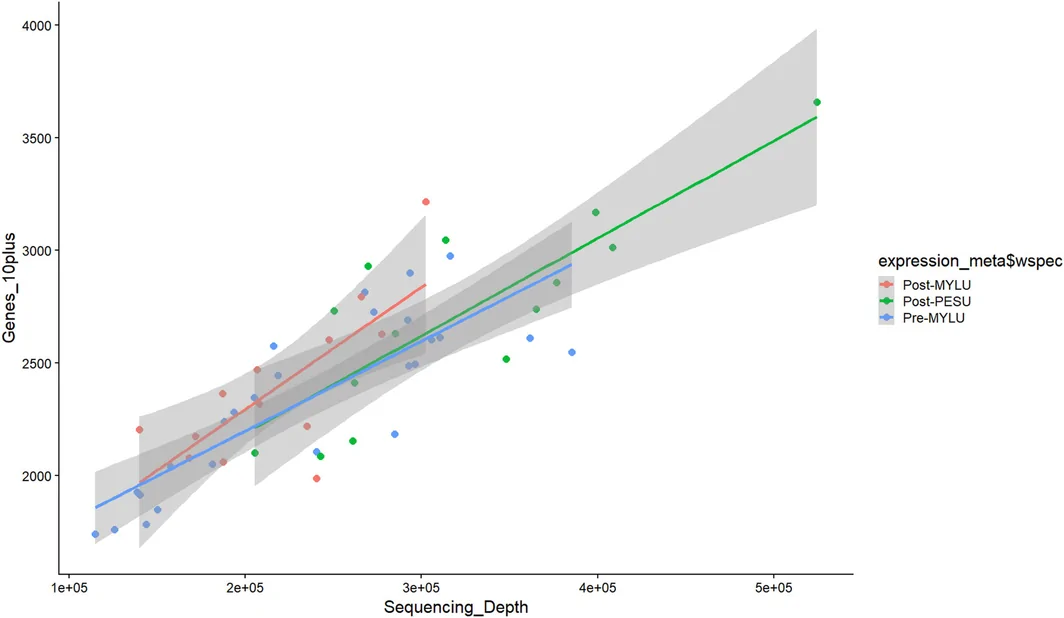
Graph of the relationship between sequencing depth and the number of discovered genes with counts greater than or equal to 10 by sample group. The lines are the estimated linear trend lines. Adjusted multiple *R* ^2^ = 0.69.

**FIGURE B3:**
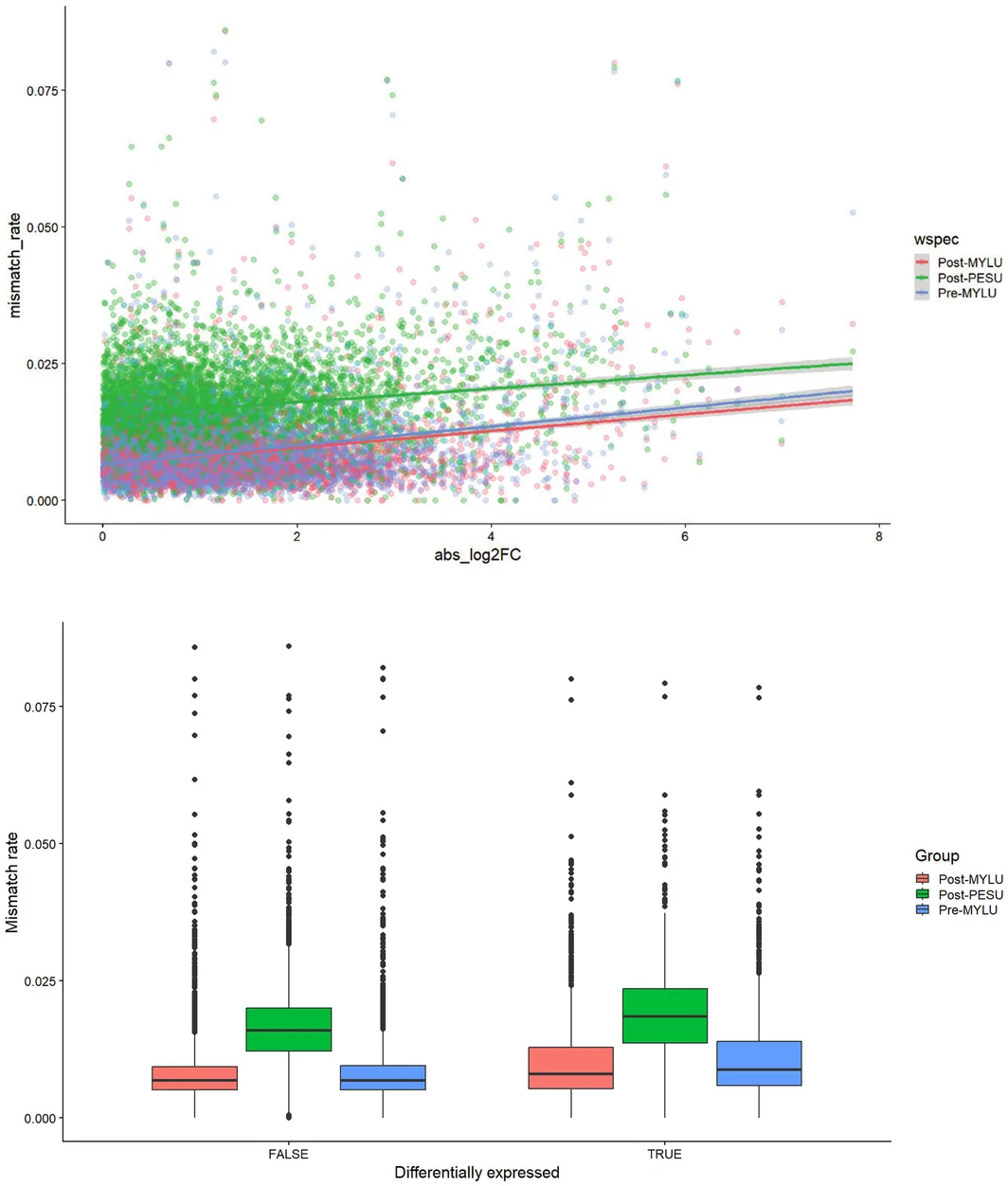
The top panel shows the graph of the relationship between per‐gene mismatch rate and the absolute value of the log_2_ FC from the analysis of post‐WNS PESU versus pre‐WNS MYLU separated by sample group. The bottom panel shows the per‐gene mismatch rate for the genes that were significantly differentially expressed in the post‐WNS PESU versus pre‐WNS MYLU comparison by sample group. Adjusted multiple *R* ^2^ = 0.30.

**FIGURE B4:**
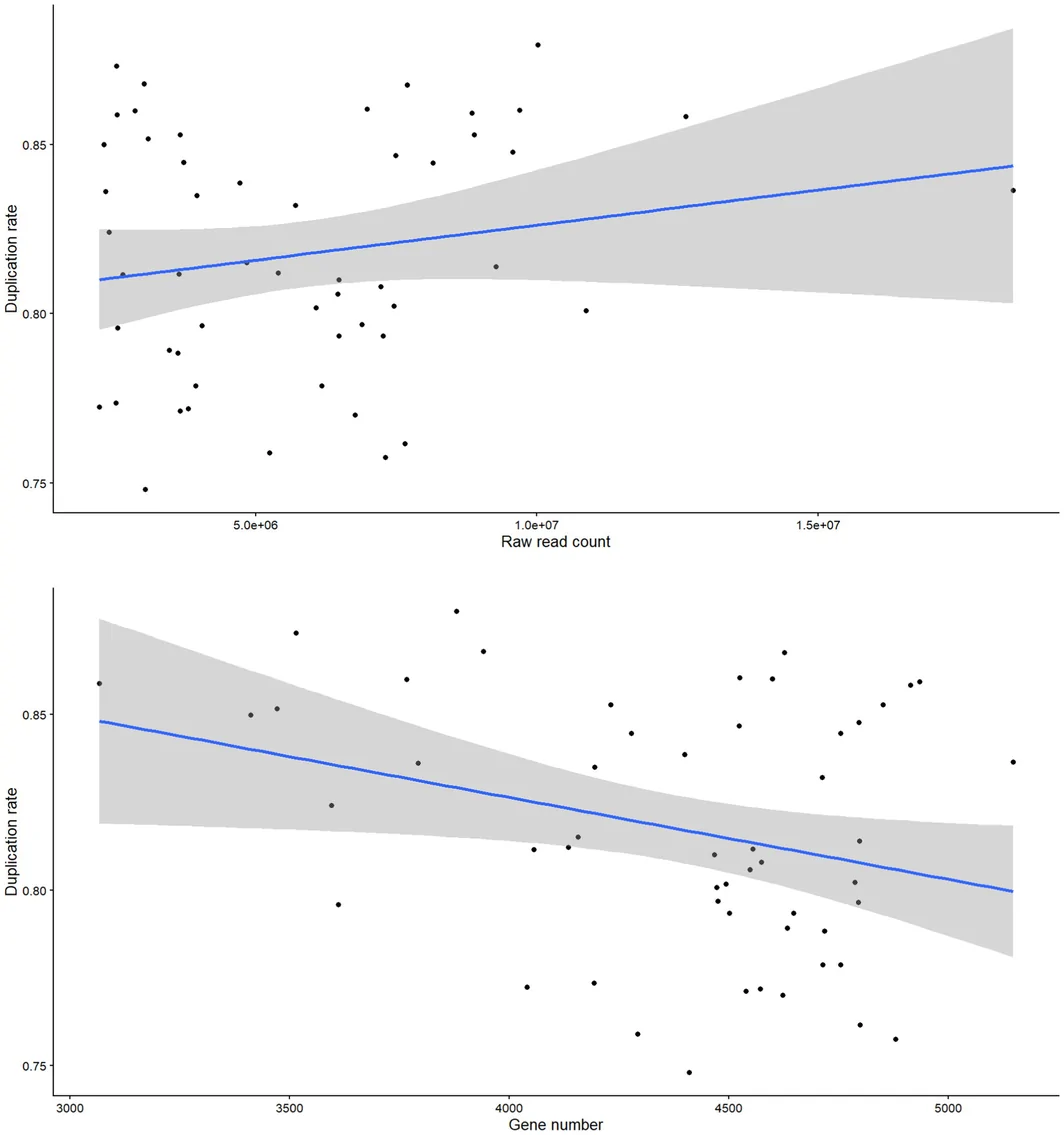
(A) The relationship between raw read count and total duplication rate calculated by *Picard*. The trend line is the model derived slope with 95% confidence intervals. Linear model coefficient = 2.07 × 10^−9^, adjusted *R* ^2^ = 0.015, *p* = 0.19. (B) The relationship between the number of genes discovered with a count > 0 and total duplication rate. The trend line is the model derived slope with 95% confidence intervals. Linear model coefficient = −2.33 × 10^−5^, adjusted *R* ^2^ = 0.071, *p* = 0.03.

**FIGURE B5:**
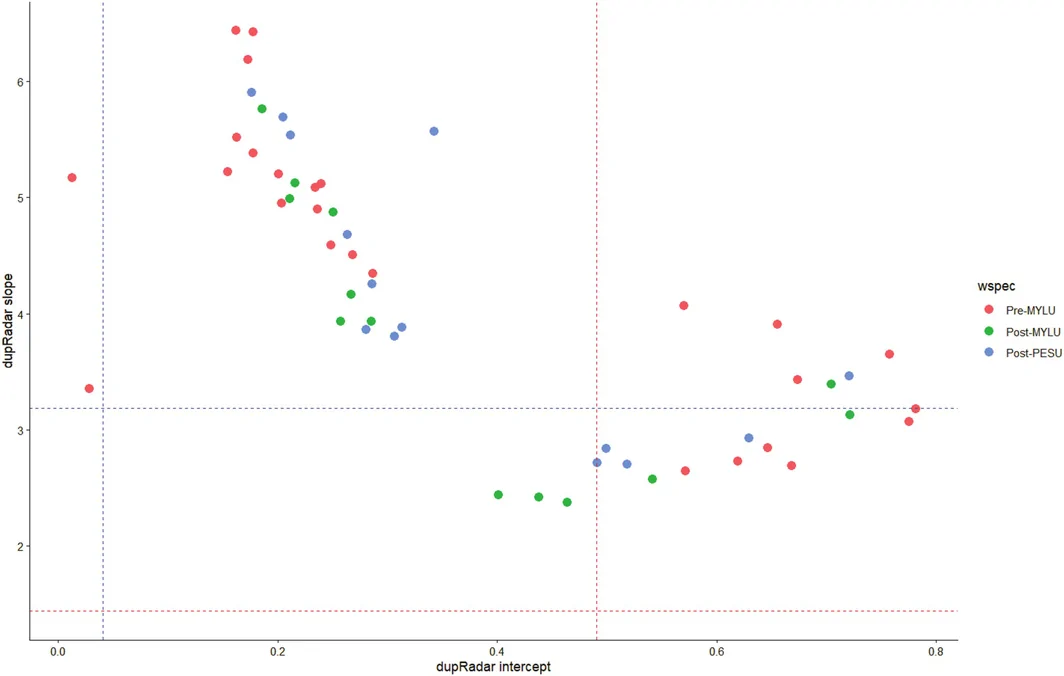
Plot showing the relationship between the intercept and slope of the lines from the *dupRadar* analysis for each sample. The blue dashed lines indicate the intercept and slope of the example “good” dataset provided by *dupRadar* (small intercept and relatively large slope) and the red dashed lines indicate the intercept and slope of the example “bad” dataset (large intercept and smaller slope).

**FIGURE B6:**
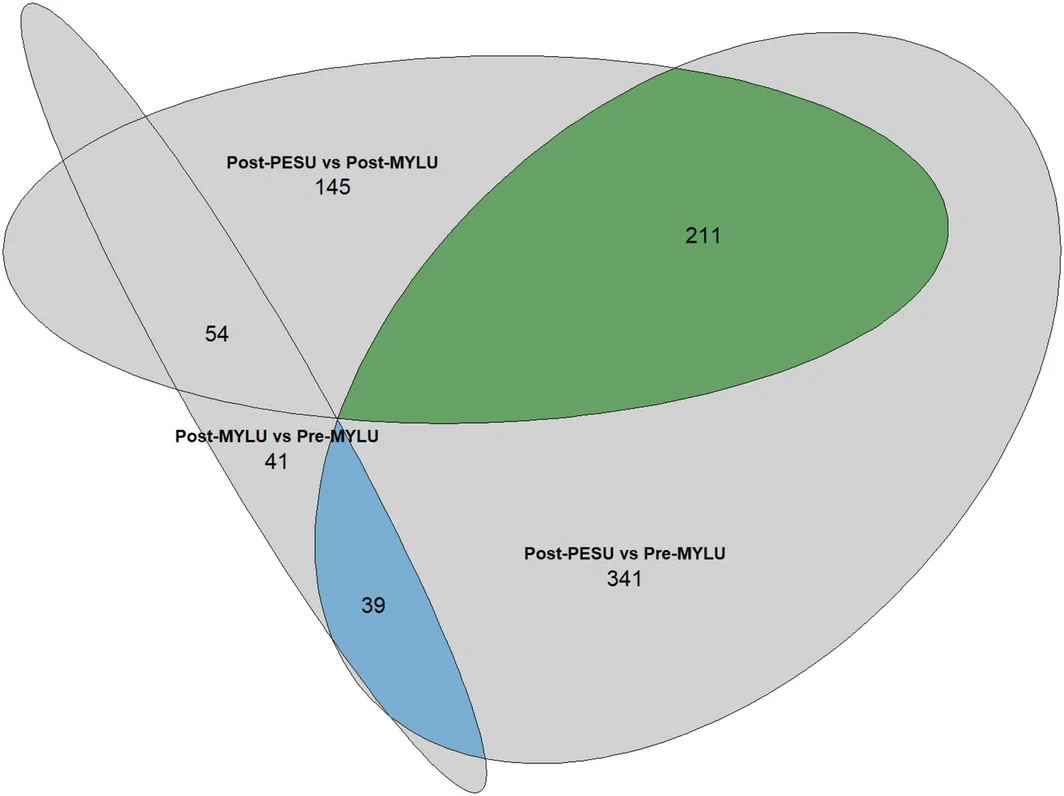
Venn diagram showing the shared and unique genes of each of three differential expression analyses using the reduced dataset. The blue shaded regions represent the regions of interest, with the overlap between the post‐WNS MYLU versus pre‐WNS MYLU and post‐WNS PESU versus pre‐WNS MYLU comparisons being considered as the putative shared response to WNS (in blue). The overlap between the post‐WNS PESU versus pre‐WNS MYLU and post‐WNS PESU versus post‐WNS MYLU comparisons is considered as consistent species level differences (in green).

**FIGURE B7:**
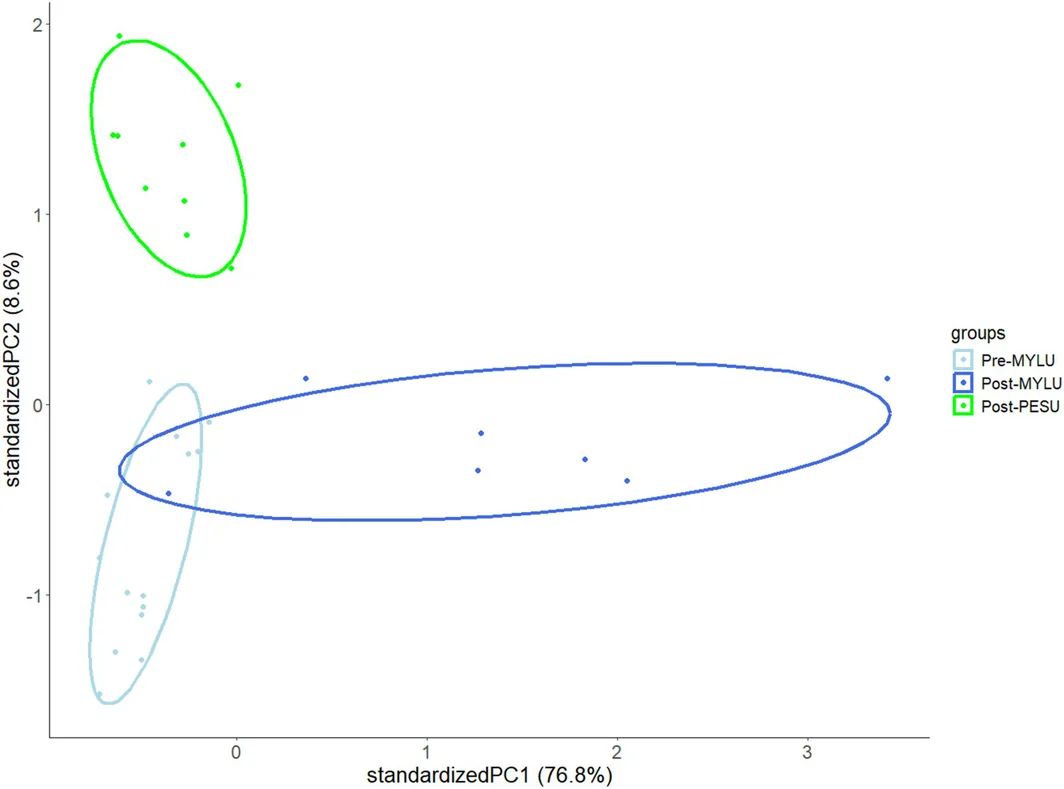
PCA biplot for the reduced dataset, excluding samples of lower quality based on the results of the *dupRadar* analysis. Colors and ellipses are by sample group.

**FIGURE C1:**
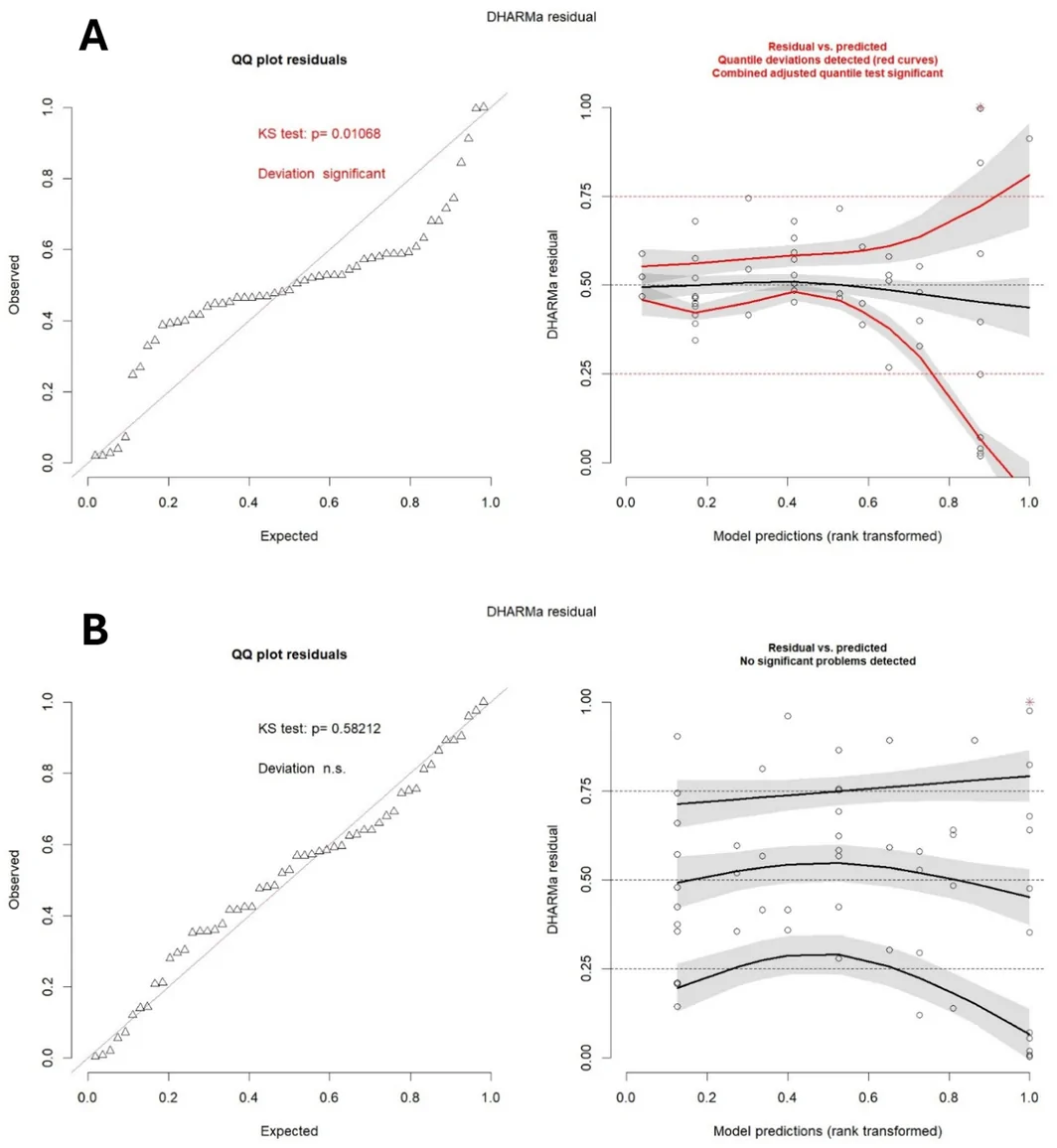
Q–Q plots and residual versus predicted plots for models including PC1 as a response variable as output by the *DHARMa* package. Panel A is for the model assuming a normal distribution and Panel B is for the model assuming a Gamma distribution with a log link function.

**FIGURE C2:**
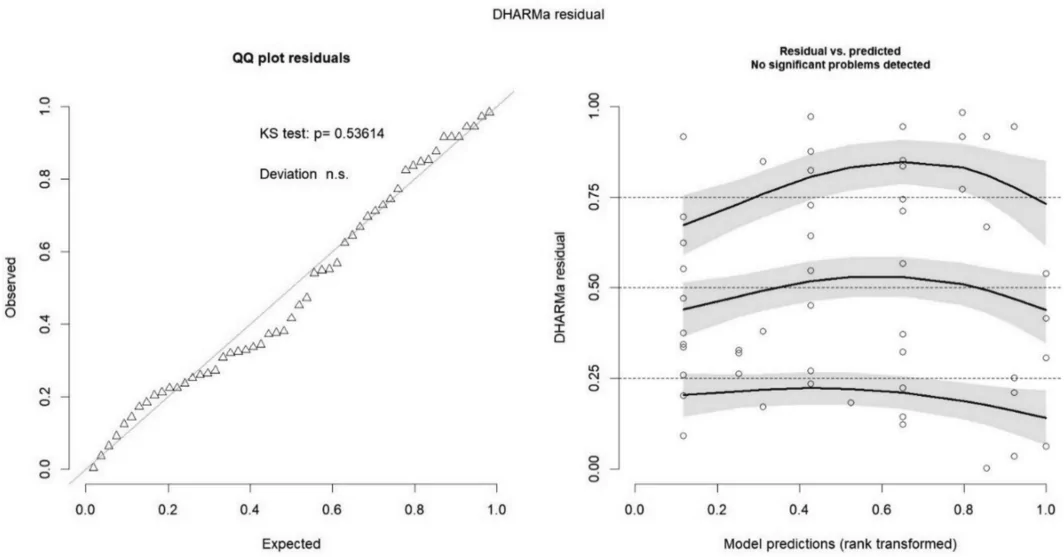
Q–Q plot and residuals versus predicted plot for model including PC2 as a response variable assuming a normal distribution as output by the *DHARMa* package.

**FIGURE D1:**
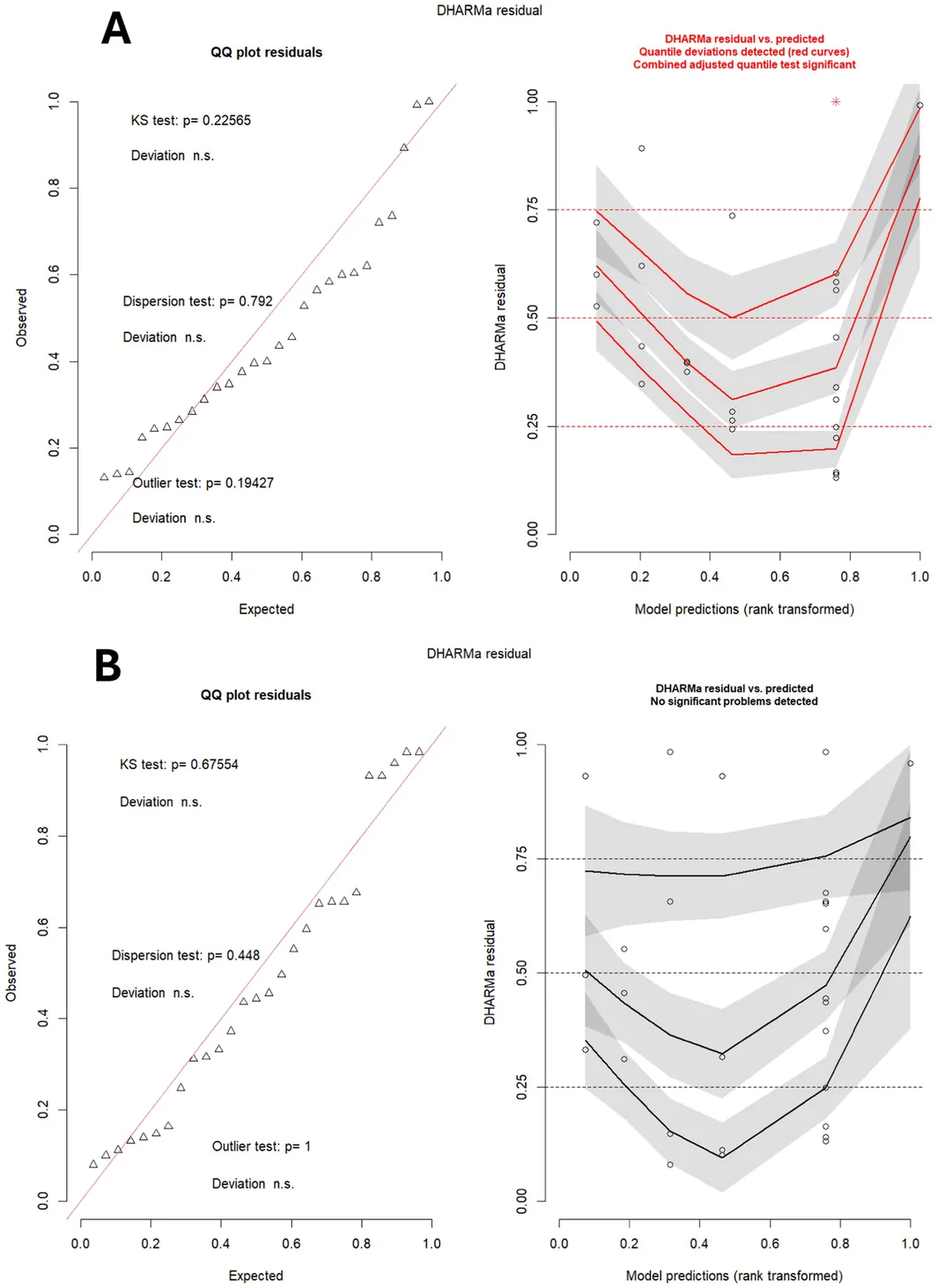
Q–Q plots and residual versus predicted plots for models of proportion of reads aligning to the *P. destructans* genome among bat species as output by the *DHARMa* package. Panel A is for the model assuming a normal distribution, and Panel B is for the model assuming a gamma distribution with a log link function.

## Data Availability

Raw sequences and metadata for all samples in this study are publicly available from the NCBI Short Read Archive. The little brown bat sequences used in this work include the late winter samples from Kentucky in the single read library of BIOProject PRJNA1115790. The tricolored bat sequences can be found in BIOProject PRJNA1545516. All code for bioinformatics and analysis can be found on GitHub (Bkrek89/Tricolored_bat_transcriptomics).

## Appendix A

**TABLE A1:** Descriptions of retained study samples, including the date of collection. The species column uses the 4‐letter acronym for each species where tricolored bat = PESU and little brown bat = MYLU. The WNS column refers to whether the samples were collected before (pre) the emergence of WNS or after the hibernaculum was exposed to WNS (post). Batch refers to the sequencing batch each sample was included in.

Collection date	Species	WNS	Site	Sex	Batch
3/26/1999	MYLU	Pre	Cave Hollow cave	M	seq2
3/26/1999	MYLU	Pre	Cave Hollow cave	M	seq2
3/26/1999	MYLU	Pre	Cave Hollow cave	M	seq2
3/26/1999	MYLU	Pre	Cave Hollow cave	M	seq2
3/26/1999	MYLU	Pre	Cave Hollow cave	F	seq2
3/26/1999	MYLU	Pre	Cave Hollow cave	M	seq2
3/26/1999	MYLU	Pre	Cave Hollow cave	M	seq2
3/26/1999	MYLU	Pre	Cave Hollow cave	M	seq2
3/26/1999	MYLU	Pre	Cave Hollow cave	F	seq2
3/26/1999	MYLU	Pre	Cave Hollow cave	M	seq2
3/26/1999	MYLU	Pre	Cave Hollow cave	F	seq2
3/26/1999	MYLU	Pre	Cave Hollow cave	M	seq2
3/26/1999	MYLU	Pre	Cave Hollow cave	M	seq1
3/26/1999	MYLU	Pre	Cave Hollow cave	M	seq1
3/26/1999	MYLU	Pre	Cave Hollow cave	M	seq1
3/26/1999	MYLU	Pre	Cave Hollow cave	F	seq1
3/26/1999	MYLU	Pre	Cave Hollow cave	F	seq1
3/26/1999	MYLU	Pre	Cave Hollow cave	F	seq1
3/26/1999	MYLU	Pre	Cave Hollow cave	F	seq1
3/26/1999	MYLU	Pre	Cave Hollow cave	F	seq1
3/26/1999	MYLU	Pre	Cave Hollow cave	F	seq1
3/26/1999	MYLU	Pre	Cave Hollow cave	F	seq1
3/26/1999	MYLU	Pre	Cave Hollow cave	F	seq1
3/26/1999	MYLU	Pre	Cave Hollow cave	F	seq1
3/26/1999	MYLU	Pre	Cave Hollow cave	F	seq1
3/26/1999	MYLU	Pre	Cave Hollow cave	F	seq1
3/16/2017	MYLU	Post	Colossal cave	M	seq2
3/16/2017	MYLU	Post	Colossal cave	M	seq2
3/16/2017	MYLU	Post	Colossal cave	F	seq2
3/16/2017	MYLU	Post	Colossal cave	M	seq2
3/16/2017	MYLU	Post	Colossal cave	M	seq2
3/16/2017	MYLU	Post	Colossal cave	M	seq2
3/16/2017	MYLU	Post	Colossal cave	M	seq2
3/16/2017	MYLU	Post	Colossal cave	M	seq2
3/16/2017	MYLU	Post	Colossal cave	M	seq2
3/16/2017	MYLU	Post	Colossal cave	M	seq2
3/16/2017	MYLU	Post	Colossal cave	M	seq2
3/16/2017	MYLU	Post	Colossal cave	M	seq2
3/16/2017	MYLU	Post	Colossal cave	M	seq2
3/16/2017	PESU	Post	Colossal cave	F	seq2
3/16/2017	PESU	Post	Colossal cave	F	seq2
3/16/2017	PESU	Post	Colossal cave	M	seq2
3/16/2017	PESU	Post	Colossal cave	M	seq2
3/16/2017	PESU	Post	Colossal cave	M	seq2
3/16/2017	PESU	Post	Colossal cave	M	seq2
3/16/2017	PESU	Post	Colossal cave	F	seq2
3/16/2017	PESU	Post	Colossal cave	F	seq1
3/16/2017	PESU	Post	Colossal cave	M	seq1
3/16/2017	PESU	Post	Colossal cave	F	seq2
3/16/2017	PESU	Post	Colossal cave	F	seq1
3/16/2017	PESU	Post	Colossal cave	M	seq1
3/16/2017	PESU	Post	Colossal cave	M	seq1
3/16/2017	PESU	Post	Colossal cave	F	seq1

## Appendix B

The results of the current study have the potential to be influenced by both reference bias and read duplication issues because; (1) we aligned tricolored bat reads to a little brown bat reference genome and (2) we used PCR amplification during library preparation with small sample inputs. Reference bias arises from sequence mismatches that result in less efficient read alignment for more distantly related species (Hekman et al. 2015; Torres‐Oliva et al. 2016). When reference bias is a concern, differential expression could be the result of actual differences in the expression of a gene or differences in alignment rate resulting in fewer copies of a gene aligning correctly (Hekman et al. 2015; Torres‐Oliva et al. 2016). Despite this concern, aligning to a closely related species can perform better than de novo transcriptome assembly (Vijay et al. 2013).

PCR duplication refers to artificial inflation of read counts due to PCR and sequencing bias. It has the potential to impact the results of an analysis if per‐gene duplication rates are not representative of true biological variation. In RNA‐Seq experiments, especially TAG‐seq, PCR duplication is difficult to properly identify and mitigate because read copies also arise as the result of actual biological variation (Williams et al. 2014; Verma et al. 2025). The current best method of addressing duplication is the employment of unique molecular identifiers (UMIs) during library preparation which allows for the tagging of copied sequences (Klepikova et al. 2017; Verma et al. 2025). In the absence of UMIs, past work generally advocates against removing duplicates for RNA‐Seq experiments because of the likelihood that the actual biological signal would be lost in doing so (Williams et al. 2014; Bansal 2017; Verma et al. 2025). However, assessment of the impact of duplication on results is warranted, especially with small input samples (Williams et al. 2014; Verma et al. 2025). Here, we assess the degree to which reference bias and duplication appear to impact our results, despite not being able to fully mitigate either factor.

### Methods

To compared the raw read counts, total duplication proportion, number of genes discovered, percentage of uniquely mapped reads, average mapped length, percentage of mismatched bases, and percentage of uncalled bases among sample groups (pre‐WNS MYLU, post‐WNS MYLU, and post‐WNS PESU). These checks compared data quality given the age and storage conditions of the pre‐WNS MYLU samples and the alignment of tricolored bat reads to a little brown bat genome. We also evaluated the relationship between the number of genes discovered and raw read count to determine if sequencing depth was sufficient for asymptotic gene discovery. We fit this as a linear model assuming a normal distribution, Michaelis‐Menton function, and a negative exponential function then compared AIC to determine which version fit best.

We then used two methods to evaluate the likelihood that reference bias influenced our results: (1) determining if the sample group had a significant impact on the relationship between sequencing depth and the number of genes with a count greater than 10 discovered, and (2) determining if the sample group had a significant impact on the relationship between per‐gene mismatch rate and the log 2 fold change (log_2_ FC) in expression after the differential expression analysis.

For the first method, reference bias has potential to impact the accumulation rate of genes discovered with sequencing depth if fewer genes map correctly in the cross‐referenced species. To test this relationship, we constructed a linear model with the number of discovered genes with a count > 10 as the response variable and the sequencing depth, sample group, and the interaction of sequencing depth and sample group as predictors. In this analysis, a significant relationship between the number of genes discovered and the interaction of sequencing depth and sample group would suggest potential reference bias.

For the second method, mismatches can lead to reduced mapping rates and thus bias genes towards significant, but completely artificial, differential expression signals (Stevenson et al. 2013; Vijay et al. 2013; Verma et al. 2025). Therefore, a significant impact of sample group on the relationship between the mismatch rate and log_2_ FC would imply that reference bias is influencing the results. We used the *Subread* package (Liao et al. 2019) and the BAM files generated by *STAR aligner* to determine the per‐gene mismatch rate. Then we combined the per‐gene mismatch rate with the differential expression analysis results to compare the absolute value of the log_2_ FC in expression for the PESU versus pre‐WNS MYLU analysis to the mismatch rate of each gene. We then constructed a linear model including mismatch rate as the response variable with the absolute value of the log_2_ FC in expression, sample group, and their interaction as predictors. In addition, we conducted an alignment with a less stringent mismatch threshold (5%) and reran the differential expression and PC regression analyses to assess the sensitivity of the analysis to mismatch leniency. The initial mismatch threshold used in the main text was 2%.

To assess whether duplication is likely to have driven our results, we first determined if total duplication rate was associated with sequencing depth or gene discovery. To that end, we constructed two linear models including total duplication rate as the response variable. One included raw read count as a predictor to determine if total duplication rate was likely to be related to over‐sequencing. The second included the number of genes discovered as the predictor, with a positive relationship indicating over‐sequencing and a negative relationship indicating sequence variation in libraries may result from input quality.

Then, we used the R package *dupRadar* to determine if the pattern of per‐gene duplication appears problematic in comparison to example datasets provided with the package (Sayols et al. 2016). First, we used *Picard* to mark duplicates in the sorted BAM files without removing duplicates. Then, we used *dupRadar* to identify the relationship between per‐gene duplication rate and reads per kilobase (RPK) of each gene for each sample. We also ran the same analysis using raw read count instead of RPK because the length of genes is not as relevant to TAG‐Seq. If duplication mirrors actual biological variation, we would expect an increase in duplication with increased gene expression, with low expression genes showing low rates of duplication and highly expressed genes showing high rates of duplication (Sayols et al. 2016). For each sample, we extracted the slope of the relationship between per‐gene duplication rate and RPK along with the intercept. Then, we plotted the slope as a function of the intercept for each sample and compared these to the intercept and slope of the examples of a “good” RNA‐Seq dataset and a “bad” RNA‐Seq dataset provided by *dupRadar*. Better libraries are expected to have a high slope and low intercept, representing lower overall duplication levels and increasing duplication with higher expression (Sayols et al. 2016). We used the large language model ChatGPT to assist in code development for the above objectives (OpenAI 2026). We thoroughly checked the resulting code.

After running *dupRadar*, we reran the *DESeq2* and PCA analyses, excluding samples with lower quality duplication patterns to assess the sensitivity of our analysis to duplication. We ran the differential expression analysis and PCA the same way we did for the full data set, including sample group as a predictor along with sex and sequencing batch as covariates. We then determined the difference in the number of genes considered differentially expressed and what percent of the differentially expressed genes from the duplication‐aware analysis were present in the original version including all samples.

### Results

Sequencing and alignment quality were broadly comparable among sample groups, although reads derived from tricolored bat samples exhibited a higher mismatch rate and shorter mapped length (Table B1). The difference in these metrics suggests we may have missed the expression of genes in tricolored bats that did not align well to the little brown bat genome. For the analysis of gene discovery as a function of raw read count, the negative exponential function (AIC = 756.4) fit better than both the Michaelis‐Menton function (AIC = 759.7) and general linear model (AIC = 776.6). Accumulation of discovered genes appeared to reach an asymptote suggesting sufficient sequencing depth (Figure B1).

There was no significant effect of sample group on the slope of the relationship between sequencing depth and the number of discovered genes with 10 or greater counts (Figure B2, Table B2). The comparison of per‐gene mismatch rate to the log_2_ FC in expression from the post‐WNS PESU versus pre‐WNS MYLU analysis showed a higher general mismatch rate in PESU samples, supporting the higher overall mismatch rate in this sample group noted above (Figure B3). The analysis also suggested that the relationship between per‐gene mismatch rate and log_2_ FC was significantly lower in PESU samples compared to MYLU samples (Figure B3, Table B3). However, the effect size was very small, expecting a −0.0003 [−0.0005, −0.0001] difference in slope of the relationship between log_2_ FC and mismatch rate between the post‐PESU samples and the post‐MYLU samples. The *p*‐value likely reflects, in part, the large number of genes included in the analysis, given the estimated difference in slopes was biologically insubstantial. In all, we did not find clear evidence that reference bias is driving our results. These analyses suggest that mismatch in the MYLU samples actually may have a stronger relationship with log_2_ FC than the PESU samples in question, which is the opposite of what one would expect if reference bias were driving the results.

Rerunning the analysis with a more lenient mismatch threshold led to broadly similar results. The PCA maintained the same structure with PC1 being significantly higher in post‐WNS MYLU than the other two groups and PC2 significantly higher in post‐WNS PESU than the other two groups. The same genes, S100A12, S100A8, and S100A4, were highly correlated with the PCs. The differential expression analysis also produced similar results with the post‐WNS PESU versus pre‐WNS MYLU, post‐WNS PESU versus post‐WNS MYLU, and post‐WNS MYLU versus pre‐WNS MYLU analyses consisting of 72%, 75%, and 75% of the same differentially expressed genes as in the original analysis. Many of the genes we focus on in the main text were maintained in the putative shared response, including but not limited to TEC, MAPKAPK3, PARP12, PGLYRP1, MIOS, and NR1D1. Thus, mismatch threshold changes create some noise in the differential expression data but do not change the overall conclusions of the analysis.

There was no significant impact of raw read count on total duplication rate (Figure B4A). There was a significant negative correlation between the number of genes discovered and duplication rate, but the effect size was small and very little variation was explained based on the *R* ^2^ (Figure B4B).

The results of the duplication analyses indicate that there are relatively high rates of duplication in this dataset, as expected from TAG‐seq. Positive slopes for every sample suggest the patterns of duplication track the patterns of expression, which is consistent with duplication being substantially associated with biological variation in gene expression rather than being largely the result of technical duplication. The intercepts of the relationships tend to be relatively high, with many falling between the good and bad example datasets from *dupRadar* and others above the bad example dataset (Figure B5). This result suggests relatively high duplication, even at low levels of expression. However, duplication is typically higher in TAG‐seq analyses than in RNA‐seq, and the *dupRadar* examples were generated from RNA‐seq. In addition, the slopes are relatively high, with most above the good example and none below the bad example. This suggests a strong relationship between expression levels and duplication rate, supporting the idea that duplication tracks expression as would be expected. The data also suggest two relatively discrete quality clusters that do not appear to be related to sample group (Figure B5). The low‐quality cluster has higher intercepts, with many crossing beyond the bad example, and low slopes. However, even this low‐quality cluster has slopes that are closer to the good example than the bad example. The higher‐quality samples have lower intercepts and higher slopes. The iteration of this analysis using the raw counts instead of RPK showed identical relationships and led to the same sample group clusters. We present the results of the iteration using RPK for simpler comparison to the *dupRadar* examples.

For a sensitivity analysis, we reran our analysis while only including samples in the higher‐quality cluster. The sample sizes for the reduced dataset were as follows: 14 pre‐WNS MYLU, 7 post‐WNS MYLU, and 9 post‐WNS PESU. The differential expression analysis resulted in 591 differentially expressed genes in the post‐WNS PESU versus pre‐WNS MYLU comparison, 410 in the post‐WNS PESU versus post‐WNS MYLU comparison, and 134 in the little brown bat response. This represents a decrease in the number of differentially expressed genes in each comparison relative to the full analysis, as expected with smaller sample sizes. Of the differentially expressed genes in the reduced analysis, 76.5% were present in the full analysis for the post‐WNS PESU versus pre‐WNS MYLU comparison, 76.1% for the post‐WNS PESU versus post‐WNS MYLU comparison, and 61.9% for the little brown bat response. The decrease in differentially expressed genes resulted in fewer shared genes in each group of comparisons, although roughly in the same proportion (Figure B6). The putative shared response in the reduced dataset consisted of 11 upregulated and 28 downregulated genes. Despite the reduced quantity, genes in the putative shared response had functions related to immune activity, the cellular starvation response, and circadian rhythms, including PGLYRP1, S100A7‐like protein, beta defensin 103, SKP1, and PSMA3 for immune genes, MIOS and SLC5A1 for metabolism, and NR1D1, LDHB, and DNM1L for circadian rhythms (Rebhan et al. 1998). These results largely mirrored our results with the full dataset.

The PCA for the reduced dataset maintained the same pattern as the PCA for the full dataset, with wide variation in PC1 for post‐WNS MYLU and separation among all three groups in PC2 (Figure B7). The same genes showed high correlation with each PC. S100A12 and S100A8 had the highest correlations with PC1 with values of 0.69 and 0.63. S100A4 had the highest correlation with PC2 with a value of 0.50. The results of the general linear model also mirrored the findings of the full analysis, with PC1 higher in post‐WNS MYLU compared to both pre‐WNS MYLU (1.77, CI [0.92, 2.61], *p* ≤ 0.0001) and post‐WNS PESU (1.67, CI [0.79, 2.54], *p* = 0.0002). There was no significant difference between post‐WNS PESU and pre‐WNS MYLU, nor were there significant relationships between PC1 and sex or sequencing batch. Post‐WNS PESU had significantly higher values of PC2 compared to both pre‐WNS MYLU (2.01, CI [1.60, 2.41], *p* ≤ 0.0001) and post‐WNS MYLU (1.84, CI [1.31, 2.38], *p* ≤ 0.0001). In contrast to the full analysis, post‐WNS MYLU did not significantly differ from pre‐WNS MYLU in PC2. Sequencing batch had a significant relationship to PC2 (0.57, CI [0.21, 0.92], *p* = 0.003) but sex did not.

In all, the analysis suggests that duplication may be a problem in this dataset for a subset of the samples. However, the patterns of association between duplication and gene expression generally support the interpretation that duplication is representative of true underlying biological variation in gene expression. The broad results of our analyses were robust even when samples with the most concerning duplication profiles were excluded. Many of the same genes are differentially expressed, and the PCA maintains the same general structure. However, the reduced dataset resulted in substantially fewer differentially expressed genes overall and introduced some that were not present in the full analysis. Part of this discrepancy likely resulted from the reduced sample size.

**TABLE B1:** Alignment and sequencing quality stats for each sample group. All values are represented as mean ± SEM Read# is the number of raw reads that passed sequencing quality filters. %Unique is the percent of reads uniquely mapped. Length refers to the average mapped length of reads aligned to the reference in base pairs. %Mismatch refers to the percentage of bases that did not match the reference. %N refers to the percentage of bases that were not identified during sequencing. Dup shows the total percent duplicates as called by *Picard*. Gene# shows the number of discovered genes with counts greater than 0 per sample.

Sample group	Read#	%Unique	Length	%Mismatch	%N	Dup	Gene#
Pre‐WNS MYLU	5,293,064 ± 548,035	78.55 ± 1.53	87.55 ± 1.11	0.89 ± 0.014	0.179 ± 0.006	0.83 ± 0.006	4214 ± 101
Post‐WNS MYLU	5,681,663 ± 722,768	69.58 ± 2.49	84.78 ± 1.69	0.97 ± 0.023	0.168 ± 0.010	0.81 ± 0.006	4507 ± 85
Post‐WNS PESU	6,911,185 ± 1,092,650	62.27 ± 1.35	65.07 ± 1.46	2.05 ± 0.105	0.155 ± 0.010	0.80 ± 0.01	4572 ± 91

**TABLE B2:** Results of analysis comparing the effect of sample group on the relationship between sequencing depth and the number of genes discovered with counts > 10. Estimate refers to the model derived coefficient. Low and High CI columns show the 95% confidence intervals of the estimate. Post‐WNS MYLU is the reference sample group. Adjusted multiple *R* ^2^ = 0.69.

Variable	Estimate	Low CI	High CI	*p*
Intercept	1211	587	1836	0.0003
Sequencing depth	0.005	0.0026	0.0082	0.0003
Post‐PESU	112.1	−686	910	0.78
Pre‐MYLU	189	−502	879	0.58
Sequencing Depth:Post‐PESU	−0.001	−0.0043	0.0021	0.49
Sequencing Depth:Pre‐MYLU	−0.001	−0.0045	0.0016	0.35

**TABLE B3:** Results of analysis comparing the effect of sample group on the relationship between the absolute value of the log_2_ FC from the post‐WNS PESU versus pre‐WNS MYLU analysis and per‐gene mismatch rate. Estimate refers to the model derived coefficient. Low and High CI columns show the 95% confidence intervals of the estimate. Post‐WNS MYLU is the reference sample group.

Variable	Estimate	Low CI	High CI	*p*
Intercept	0.007	0.006	0.007	< 0.001
Abs(log_2_ FC)	0.002	0.001	0.002	< 0.001
Post‐PESU	0.009	0.009	0.009	< 0.001
Pre‐MYLU	−0.00001	−0.0004	0.0004	0.95
Abs(log_2_ FC):Post‐PESU	−0.0003	−0.0005	−0.0001	0.01
Abs(log_2_ FC):Pre‐MYLU	0.0002	−0.00002	0.0004	0.07

## Appendix C

Model quality control for the regression of principal component 1 suggested problems with dispersion and residual quantile similarity leading to poor model fit with a normal distribution (Figure C1A). Employing a generalized linear model assuming a Gamma distribution with a log link function significantly improved model fit (Figure C1B). However, the assumed distribution of the model had no influence on interpretation, so we included the results of the model assuming a normal distribution in the main text. Sample group was still the only significant variable (Table C2), and pairwise comparison still showed significant differences between post‐WNS MYLU and the other two groups (Table C3). The regression of principal Component 2 showed acceptable model fit with a normal distribution (Figure C2).

**TABLE C1:** Full model results from the principal component regression. Model set notes which principal component was the response variable. Estimate refers to the model derived coefficient with 95% confidence intervals.

Model set	Variable	Estimate	*p*
PC1	Intercept	1.030 [0.303, 1.757]	0.007
PC1	Group (Post‐PESU)	−1.353 [−2.013, −0.693]	0.0002
PC1	Group (Pre‐MYLU)	−1.469 [−2.086, −0.853]	< 0.0001
PC1	Sex (M)	−0.049 [−0.557, 0.459]	0.849
PC1	Batch	0.124 [−0.409, 0.657]	0.647
PC2	Intercept	−0.262 [−0.582, 0.058]	0.112
PC2	Group (Post‐WNS PESU)	1.660 [1.369, 1.950]	< 0.0001
PC2	Group (Pre‐WNS MYLU)	−0.568 [−0.839, −0.297]	0.00013
PC2	Sex (M)	0.067 [0.156, 0.291]	0.553
PC2	Batch	0.101 [−0.134, 0.335]	0.399

**TABLE C2:** Full model results for PC1 model assuming a Gamma distribution. Estimate refers to the model derived coefficient with 95% confidence intervals.

Variable	Estimate	*p*
Intercept	0.531 [0.111, 0.950]	0.016
Group (Post‐WNS PESU)	−0.969 [−1.350, −0.588]	< 0.0001
Group (Pre‐WNS MYLU)	−1.165 [−1.521, −0.809]	< 0.0001
Sex (M)	0.052 [−0.241, 0.345]	0.727
Batch	0.170 [−0.138, 0.477]	0.28

**TABLE C3:** Model results for pairwise comparisons of principal component 1 among sample groups assuming a Gamma distribution. Query shows which group is being tested and reference shows which group is being considered a reference. Estimate refers to the model‐derived coefficient with 95% confidence intervals. The *p*‐value is the Tukey adjusted *p*‐value for pairwise comparisons.

Model set	Query	Reference	Estimate	*p*
PC1	Post‐MYLU	Post‐PESU	0.969 [0.589, 1.349]	< 0.0001
PC1	Post‐MYLU	Pre‐MYLU	1.165 [0.809, 1.521]	< 0.0001
PC1	Post‐PESU	Pre‐MYLU	0.196 [−0.107, 0.499]	0.415

## Appendix D

For the comparison of relative *P. destructans* load between species, the model assuming a normal distribution showed poor model fit (Figure D1A). Employing a Gamma istribution ith a og ink unction mproved odel it (Figure D1B). Both versions of the model suggested that tricolored bats had significantly lower proportions of reads aligning to *P. estructans* (Table D1).

**TABLE D1:** Results for models of proportion of reads aligning to *P. destructans* among species of bat. Distribution shows which distribution the model assumed. The model assuming a Gamma distribution was run as a generalized linear model with a log link function. Estimate shows the coefficient with 95% confidence intervals.

Distribution	Variable	Estimate	*p*
Normal	Intercept	2.99 × 10^−6^ [1.45 × 10^−7^, 5.84 × 10^−6^]	0.0403
Normal	Species (PESU)	−2.22 × 10^−6^ [−4.09 × 10^−6^, −3.43 × 10^−7^]	0.0224
Normal	Sex (M)	−7.87 × 10^−7^ [−2.59 × 10^−6^, 1.02 × 10^−6^]	0.3774
Normal	Batch	7.54 × 10^−7^ [−1.30 × 10^−6^, 2.81 × 10^−6^]	0.4553
Gamma	Intercept	−13.78 [−14.96, −12.52]	< 0.0001
Gamma	Species (PESU)	−0.98 [−1.76, −0.16]	0.0325
Gamma	Sex (M)	−0.02 [−0.81, 0.69]	0.9548
Gamma	Batch	1.08 [0.12, 2.01]	0.0313
